# The NCATS BioPlanet – An Integrated Platform for Exploring the Universe of Cellular Signaling Pathways for Toxicology, Systems Biology, and Chemical Genomics

**DOI:** 10.3389/fphar.2019.00445

**Published:** 2019-04-26

**Authors:** Ruili Huang, Ivan Grishagin, Yuhong Wang, Tongan Zhao, Jon Greene, John C. Obenauer, Deborah Ngan, Dac-Trung Nguyen, Rajarshi Guha, Ajit Jadhav, Noel Southall, Anton Simeonov, Christopher P. Austin

**Affiliations:** ^1^Division of Pre-Clinical Innovation, National Center for Advancing Translational Sciences, National Institutes of Health, Rockville, MD, United States; ^2^Rancho BioSciences, San Diego, CA, United States

**Keywords:** BioPlanet, pathway, systems biology, chemical genomics, *in vitro* assay

## Abstract

Chemical genomics aims to comprehensively define, and ultimately predict, the effects of small molecule compounds on biological systems. Chemical activity profiling approaches must consider chemical effects on all pathways operative in mammalian cells. To enable a strategic and maximally efficient chemical profiling of pathway space, we have created the NCATS BioPlanet, a comprehensive integrated pathway resource that incorporates the universe of 1,658 human pathways sourced from publicly available, manually curated sources, which have been subjected to thorough redundancy and consistency cross-evaluation. BioPlanet supports interactive browsing, retrieval, and analysis of pathways, exploration of pathway connections, and pathway search by gene targets, category, and availability of corresponding bioactivity assay, as well as visualization of pathways on a 3-dimensional globe, in which the distance between any two pathways is proportional to their degree of gene component overlap. Using this resource, we propose a strategy to identify a minimal set of 362 biological assays that can interrogate the universe of human pathways. The NCATS BioPlanet is a public resource, which will be continually expanded and updated, for systems biology, toxicology, and chemical genomics, available at http://tripod.nih.gov/bioplanet/.

## Introduction

For most of its history, the field of toxicology has focused predominantly on whole-organism studies, with observable histological, behavioral, or developmental endpoints, or “apical endpoints,” being cataloged as occurring after exposure to chemicals. While whole-organism studies have served as the backbone of scientific and regulatory imperatives to protect human health, they suffer from lack of mechanistic insights, high cost, low throughput, and uncertain applicability to human risk assessment. However, unlike systems pharmacology and drug development, toxicology assessment has changed relatively little in the last 50 years (Kavlock et al., 2009; Hamburg, 2011) due, in part, to the regulatory context in which most toxicological assessment takes place, and the human bias that (only) “seeing is believing.”

An example of a recently initiated effort to explore *in vitro* approaches to toxicology, the United States Tox21 program (National Research Council [NRC], 2007) was constituted in 2007 to utilize high-throughput *in vitro* testing and computational methods to transition toxicology into a predictive, mechanistic science (Collins et al., 2008; Kavlock et al., 2009; Tice et al., 2013). A collection of approximately 10,000 drugs and environmental chemicals (Attene-Ramos et al., 2013b) has been tested at 15 concentrations using a robotic platform (Inglese et al., 2006) in a wide variety of assays (Huang et al., 2016) with the initial focus on stress-response (Attene-Ramos et al., 2013a; Nishihara et al., 2015) and nuclear hormone receptor pathways (Hsu et al., 2014; Huang et al., 2014). However, given the protean nature of toxicological endpoints, and the lack of understanding of the molecular mechanism(s) that lead to most of these endpoints, characterization of the chemicals’ effects in a much broader set of assays will be required. Ideally, a set of assays could be selected or designed to measure targets that encompass all pathways that are relevant to toxicity. However, what constitutes a “toxicity pathway” is not clearly defined. A recent report (National Research Council [NRC], 2007) states that “toxicity pathways” are “cellular response pathways that, when sufficiently perturbed in an intact animal, are expected to result in adverse health effects.” This definition could potentially refer to all biological pathways, as our current understanding of the biological system is not sufficient for us to pinpoint the specific subset of pathways that fit this description. Molecular pathways are defined not only by their importance in normal physiology, but also by the disease or adverse events caused by their dysfunction. Since toxicological endpoints may potentially be caused by dysfunction of any pathway operative in human cells, mechanistic understanding and predictive signatures for all endpoints may ultimately require profiling of the Tox21 and/or other chemicals in a suite of assays that encompass all human pathways, representing a highly implausible scenario.

As a first step to enabling this goal, we aimed to develop a complete and non-redundant catalog of all human pathways, and construct an informatics platform to represent and browse the pathways, their healthy and disease state annotations, and targets within and relationships among them at varying levels of detail. Such a platform would enable the rational construction of a minimal set of assays that could be used to query all of pathway space experimentally, given that many pathways overlap and together form a network subsuming all cellular functions. Toward this goal, this platform can serve as a starting point for the systematic design of experiments to better understand how biological systems function. When linked with bioactivity data, the pathway data can be used to examine and predict the network effects of chemicals and other perturbations. Such a public resource would not only be critical to fulfilling the goals of *in vitro* toxicology efforts, but also provide fundamental values to the biomedical research community as a whole.

Existing pathway databases tend to focus on particular areas of biology, e.g., metabolism vs. signaling, and a comprehensive and uniform resource that covers all known pathways and their annotations does not exist (Galperin and Cochrane, 2011; Galperin and Fernandez-Suarez, 2012). Moreover, information in many databases are computationally generated, e.g., HumanCyc^1^, and not derived from direct experimental evidence, which is generally deemed more reliable. Other efforts that attempt to integrate individual resources, e.g., Pathway Commons (Cerami et al., 2011) simply combine data from various databases without further curation or validation of the information collected to remove redundancy or improve data quality. Different types of data are often mixed together with no distinctions made between, e.g., pathways and protein–protein interactions, experimental results and computational predictions, and no additional annotations are provided. Commercial pathway resources and tools are claimed to be more comprehensive (e.g., Ingenuity, GeneGo) (Thomas and Bonchev, 2010) yet the access by the research community to these products is hampered by the high cost. Our aim is to develop an open-source solution to enable researchers worldwide to access the tools and the data without encumbrance.

**FIGURE 1:**
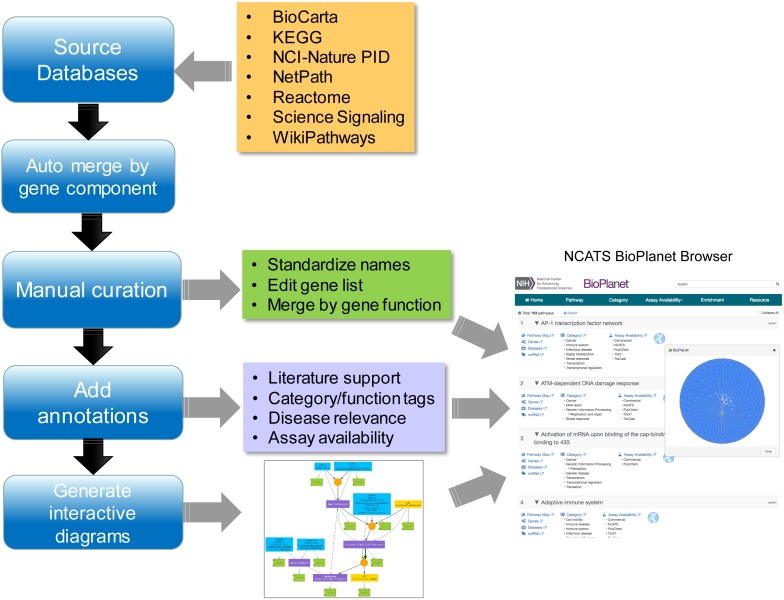
Workflow for the NCATS BioPlanet construction process.

We report here the construction, features, and utilization of a comprehensive integrated and non-redundant pathway resource, the NCATS BioPlanet (Figure 1). The resource hosts information only from public sources that have been herein further manually curated to ensure the quality of the data. Along with our pathway warehouse, the NCATS BioPlanet software platform allows easy browsing and visualization of the universe of pathways, and exploration of associations among them. Additionally, we curated the set of annotated pathways in terms of the biological space covered and the current availability of assays, either commercial or academic, to probe each subspace. After eliminating redundancy across the pathway databases used to create the BioPlanet, we found that human cells incorporate 1,658 pathways. Starting with these pathways, we utilized a condensation approach to construct a minimal set of assays to cover all of pathway space. This minimal set of pathways will serve as the starting point to prioritize pathways for testing in a wide variety of systems biology efforts, and provides a reduced-complexity set for the systems pharmacology community. The NCATS BioPlanet will be continually updated and is publicly accessible at http://tripod.nih.gov/bioplanet/.

## Data, Methods, and Results

### Source Databases

Annotations for pathways and gene-gene or protein–protein interactions were obtained from a number of publically available databases, in which pathway annotations are also manually generated based on experimental observations to ensure the quality of our data sources. The locations and contents of these databases are listed in Table 1. Annotations of human disease genes were downloaded from the Online Mendelian Inheritance in Man (OMIM) database (McKusick, 1998). Gene target information for assays was extracted from PubChem bioassay descriptions (PubChem, 2010).

**Table 1:** Pathway data sources.

Database	Number of human pathways	Number of genes	URL
KEGG – Kyoto Encyclopedia of Genes and Genomes	214	5520	http://www.genome.jp/kegg/pathway.html
BioCarta^∗^	314	1494	https://cgap.nci.nih.gov/Pathways/BioCarta_Pathways
Reactome - a curated knowledgebase of biological pathways	1283	6125	http://www.reactome.org/
WikiPathways	204	4064	http://www.wikipathways.org/
NCI-Nature – Pathway Interaction Database^∗^	722	3725	https://www.nlm.nih.gov/research/umls/sourcereleasedocs/current/NCI_PID/
Science Signaling^∗^	58	1234	http://stke.sciencemag.org/about/help/cm
NetPath	35	2877	http://www.netpath.org/

*^∗^Original database site is no longer supported. The URL provided here points to some data hosted at an alternative site.*

The present study focused on pathways annotating human genes. Different pathway sources focused on different aspects of the human biological system. KEGG is a large pathway database annotating over 5,500 human genes with a heavy focus on metabolism (KEGG, 2010). Over 50% of the KEGG pathways are metabolic pathways with the second largest pathway category, human diseases, making up only 14% of all KEGG pathways. The Science Signaling database (support ended in 2015) (Science Signaling, 2010) as its name indicates, is a collection of cell signaling pathways. Its pathway maps were generated based on information provided by scientists with expertise in a given field, deemed “pathway authorities,” thus assuring the quality of the data. A result of collaborative efforts between the National Cancer Institute (NCI) and the Nature Publishing Group, the NCI-Nature Pathway Interaction Database (PID) (now retired) (NCI-Nature, 2010) is another source of curated human signaling pathways. Reactome is an open-source, curated pathway database that covers a variety of human biology including cell signaling, metabolism, human diseases and other fundamental biological processes, with some emphasis on signaling and metabolic pathways, comprising 18% and 17% of the pathways, respectively (Reactome, 2010). BioCarta pathway collection (no longer supported) operated as an open-source, community-fed forum with annotations collected on over ten different biological functions and processes, but with cell signaling as the primary category encompassing 32% of all BioCarta pathways (BioCarta, 2010). Similar to BioCarta, WikiPathways adopts the open source approach, as well, which takes input from the scientific community for the curation of biological pathways (WikiPathways, 2010). WikiPathways annotates over 4,000 human genes encompassing a range of pathway categories, including signaling (∼30%) and metabolic (∼10%) pathways.

### Removing Redundancy

**FIGURE 2:**
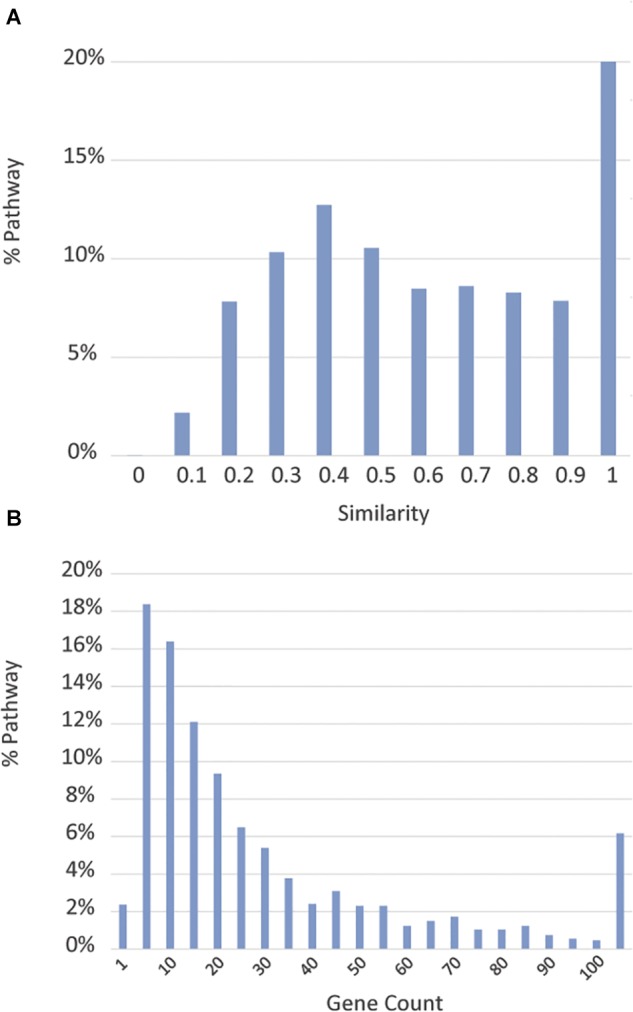
Overview of source pathways. **(A)** Distribution of similarity scores, measured by the degree of gene component overlap, of pathways from different databases. **(B)** Distribution of pathway sizes measured by gene count.

As expected, we found substantial overlaps among the pathway databases. To assess the extent of redundancy, we calculated a similarity score, defined as the ratio of genes shared between two pathways over the total number of unique genes contained in the two pathways, between each pathway and the pathway with which it has the highest gene component overlap. Figure 2A shows the distribution of these similarity scores. Approximately 23% of the pathways have at least one complete duplicate with identical gene components, and 31% of the pathways have at least one close match, with which they share over 90% of genes, in a different data source. Moreover, many pathways have only a few genes annotated. As shown in Figure 2B, about 20% of the pathways have ≤5 genes and 2.4% of the pathways only have one gene. Annotation of these pathways thus appears to be incomplete. For ease of downstream analysis, we chose to merge pathways with no significant difference in their gene components and exclude pathways with less than three genes whenever appropriate to minimize redundancy (see below for the procedure details). Utilizing these criteria, we found that there are 1,658 distinct pathways, encompassing 9,818 human genes, which constitute approximately 40% of all human genes. The number of pathway genes and the details of their relationships can be reasonably expected to change as functions of more genes are discovered and their interactions elucidated. Therefore, the content of the BioPlanet will be curated and updated periodically to reflect the updates from our data sources and to incorporate information from any new data sources that might emerge. As this project is constantly evolving, mistakes and incompleteness are inevitable and we have set up a mechanism for the scientific community to send us feedback and corrections to improve the quality of the BioPlanet as a public resource.

Two pathways were merged into one by merging their gene components when one of four criteria was met: (1) the overlap, defined as the number of genes shared by the two pathways divided by the total number of unique genes in the two pathways, was >90%; (2) the two pathways differ by only one gene; (3) one pathway has <3 genes and all of these genes are contained in the other pathway; (4) the two pathways have >50% overlap in their gene components and *p* < 0.05 (Fisher’s exact test). The merging procedure was repeated until no two pathways met any of these criteria. After merging, the pathway gene lists were manually curated to correct mis-assigned genes and further remove redundancy (Figure 1; see below for detailed curation procedure) resulting in a final list of 1,658 distinct pathways.

### Extensive Manual Curation

After initial merging, BioPlanet included 1,774 pathways that contained 10,040 unique genes, 9,928 of which were assigned to *Homo sapiens*. The pathway names were standardized and corrected for consistent capitalization, biological clarity, usage of Greek letters, and hyphenated terms. The genes in the pathways were also edited to remove withdrawn identifiers and replace obsolete ones. Non-human genes were removed or replaced with corresponding human genes. However, even after the removal of non-human genes, a few pathways from mice and other species remained. Yet, despite the pathway names, all genes in these pathways were human. Therefore, to ameliorate this discrepancy, these pathways were renamed to remove the animal inference. Extremely small pathways that contained only one or two genes were merged with larger pathways, and some pathways with similar names and functions were merged. Eighty-nine sets of pathways had very similar names but different gene lists. For example, “Alzheimer’s disease” has 168 genes, and a separate pathway called “Alzheimer’s disease” has 82 genes, but some genes from the latter set are not among the genes in the former one. To resolve similarly named pathway sets like these, each set was manually examined to establish whether their gene lists had sufficiently similar functions to substantiate merging of these pathways, or whether the functions were sufficiently different, and the pathways should have been kept separate under distinctly different names. To make these decisions, the genes unique to each pathway were uploaded to the DAVID annotation resource^2^. Using DAVID’s Functional Annotation Clustering tool, the top annotation cluster characterizing the gene list was used to determine the collective function of these genes. Gene Ontology Biological Process terms, KEGG pathways, and BioCarta pathways were preferred when available. Based on these results, pathway sets with gene lists that had sufficiently similar functions were merged. The pathways with gene lists that had distinct functions were preserved as separate pathways and renamed to distinguish them better. In total, 714 of the 1,774 pathway names (40%) were edited. Some pathways were removed or merged with other ones during the process, reducing the total number of pathways to 1,658. The number of unique genes represented in the pathways was reduced from 10,040 to 9,818.

Literature supporting the pathways and interactions were first added computationally. For the 303 pathways with no literature association found through the automated approach, references were sourced manually. GeneRif^3^ was used to link genes to literature references (PubMed IDs). Pathway names and gene lists were used to search PubMed to find pathway-literature linkages. PubMed IDs shared between genes and pathways were then identified to establish the gene-pathway association. An average of 50 abstracts from each method were spot-checked to ensure the method was producing the correct results. A total of 234,347 unique references were found for all 1,658 pathways, with each pathway having at least one reference. Further curation of the gene–gene interactions within each pathway is currently underway. Publications supporting the interactions selected by the pathway authors are retrieved from the source files and added to the BioPlanet pathways.

### Pathway Tagging

Keyword tags were used to group functionally related pathways into categories. The GO Slim biological processes^4^, a small set of high-level functions characterizing an organism, were used to generate the list of pathway tags. Some GO Slim terms were rejected for being too long (“anatomical structure formation involved in morphogenesis”), only applying to one pathway (“ribosome biogenesis”), or describing processes that do not exist in humans (“photosynthesis”). Disease-related tags were added based on the top-level disease categories at Disease Ontology^5^. Tags used by the source databases to group pathways were also collected for inclusion. Redundant tags were removed or merged with existing tags. A total of 51 tags were eventually selected, and grouped into seven categories: Major Systems, Cell Cycle, Genetic Information Processing, Metabolism, Development, Signaling, and Disease. The tags in each category are listed in Table 2.

**Table 2:** Pathway tags.

Major systems	Metabolism	Signaling
Circulatory system	Nucleic acid metabolism	Cell signaling
Digestive system	Carbohydrate metabolism	G-protein coupled receptor
Endocrine system	Protein metabolism	Nuclear receptor
Excretory system	Lipid metabolism	Transcriptional regulation
Immune system	Vitamin and cofactor metabolism	Stress response
Musculoskeletal system	Small molecule metabolism	Environmental adaptation
Nervous system	Xenobiotic metabolism	Chronology
Sensory system	Energy metabolism	Transport
		
**Genetic information processing**	Protein folding, sorting, and degradation	
DNA replication	Protein modification	**Disease**
DNA repair		Cancer
Transcription		Cardiovascular disease
RNA processing	**Cell cycle**	Genetic disease
Translation	Cell cycle	Immune disease
	Cell growth	Infectious disease
**Development**	Cell death	Neurological disease
Development	Cell division	Physical disorder
Adhesion	Cell proliferation	Endocrine and metabolic disease
Cell differentiation	Reproduction	Sepsis
Cell motility		Substance dependence

GO annotations for human genes were used to tag many of the pathways automatically. The tag keywords were first matched manually to GO terms in the top 4 levels of the GO hierarchy. For each GO term, up to three tags were assigned. Most level-4 terms were not manually tagged unless they also occurred in a higher level. These terms were then associated with genes using the GO annotations, and the gene lists for each pathway were used to determine whether enough genes with one tag were present to assign that tag to the whole pathway. Specifically, we required that (1) at least 10% of the genes in the pathway have the same tag and (2) at least four genes have the same tag. The automated GO term method assigned at least one tag to 84% of the pathways. However, the GO term method missed some obvious tags suggested by the pathway titles. For example, “HIV-induced T cell apoptosis” would be expected to get tags for “Infectious disease” (“HIV” in the name), “Immune response” (“T cell”), and “Cell death” (“apoptosis”). For this reason, a second component was added to the automatic tagging algorithm. A list of keywords was created that would be expected to match each tag, and the occurrence of these keywords in the pathway title would assign the corresponding tags. For example, the tag “Nucleic acid metabolism” would be assigned if the pathway title contained words like “Nucleobase,” “Nucleotide,” “Nucleoside,” “Purine,” or “Pyrimidine.” This keyword method assigned at least one tag to 58% of the pathways. The combination of the two methods yielded 92% pathways with at least one tag. To measure how well the automated tagging process worked, 10 pathways were selected for manual review. The results showed that the automated process produced a high false positive (63%) and low false negative rate (30%). For this reason, we decided to manually review all of the tagged pathways, removing tags that seemed irrelevant and adding tags that were missed.

A manual workflow was then applied to add missing tags, remove false positive tags, and add disease tags to pathways. For each pathway, one or more summaries of the pathway were found from online scientific sources like PubMed, Entrez Gene (Maglott et al., 2005) and BioCarta, and the decisions to add or remove tags were based on these summaries. The Comparative Toxicogenomics Database (CTD^6^) was used to find relevant disease associations. The list of gene IDs from the pathway was entered into CTD’s gene set analyzer and disease Venn diagram. The first method shows a list of diseases associated with the input gene set, ranked by *p*-value, and the second method shows the overlap between the input gene set and the disease gene set. Rather than relying on a *p*-value threshold or minimum number of genes, high-ranking diseases in the list were accepted if they were consistent with the pathway description. This prevented the problem we observed in some of the automated tag assignments, when, for example, a subset of pathway genes may be involved in an Infectious Disease but the corresponding pathway is not primarily associated with any such Infectious Disease. After manual curation, all pathways have at least one tag assigned. The median number of tags per pathway is 5, while the maximum number is 15.

### Assay Availability for Pathway Interrogation

**FIGURE 3:**
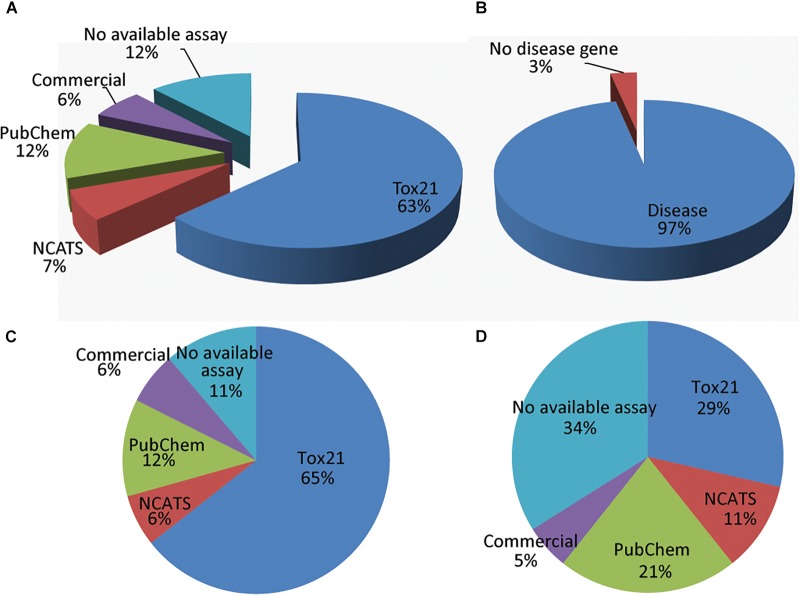
Disease pathways and assay availability from four sources (in the order of decreasing priority): (1) Tox21, (2) NCATS, (3) PubChem, and (4) commercial vendors. If a pathway was covered by assays from multiple sources, only the source with the highest priority was counted. **(A)** Assay availability for all pathways. **(B)** Fraction of pathways that are disease pathways. **(C)** Assay availability for disease pathways. **(D)** Assay availability for non-disease pathways.

Since one rationale for creating the BioPlanet is to enable the experimental assessment of chemical modulation of a wide range of human pathways, we next explored the current availability of extant bioassays to probe the 1,658 distinct human pathways. We examined bioassays from four sources, which cover 2,685 gene targets in total (in the order of decreasing priority): (1) assays from the Tox21 program that have been run at NCATS, (2) other NCATS assays, (3) other bioassays in PubChem, and (4) assays from commercial vendors not yet employed by Tox21, NCATS, or PubChem assay providers. Phenotypic assays with no specific gene targets were excluded from the analysis. Figure 3A shows the coverage of the 1,658 human pathways by assays from these four sources. If a pathway was covered by assays from multiple sources, only the source with the highest priority was counted. For example, if an assay was available from both Tox21 and PubChem, the pathway would be counted as covered by Tox21 in Figure 3A (see Supplementary Figure S1 for the coverage of the BioPlanet pathways by each individual source). All available assay sources for each pathway can be found in the BioPlanet database and browser. Here, to get an initial estimate, we have not made a distinction between assays that measure a specific gene target in a pathway, and pathway assays, i.e., assays that measure signaling throughout that pathway. We found that 88% of the pathways have at least one gene target with an assay available from one of the four assay sources, and 12% of the pathways do not have a bioassay available from these sources (Supplementary Figure S2). Of the four sources, the Tox21 assays cover 63% of the pathways; when combined with the other NCATS assays, these two sources cover 70% of the 1,658 pathways. Assays from other PubChem assay providers cover 12% of the pathways and we found other commercial assays for another 6% of the pathways. Recent developments in the field of precision medicine and RNA based therapeutics have highlighted the role of non-coding RNA (ncRNA) (Cech and Steitz, 2014) such as lncRNA (Volders et al., 2019), miRNA (Chou et al., 2018), and circRNA (Glazar et al., 2014) in healthy and disease conditions. When annotated by the availability of non-coding RNAs, we found that >99% of the BioPlanet pathways are regulated by at least one ncRNA (Supplementary Figure S1).

**FIGURE 4:**
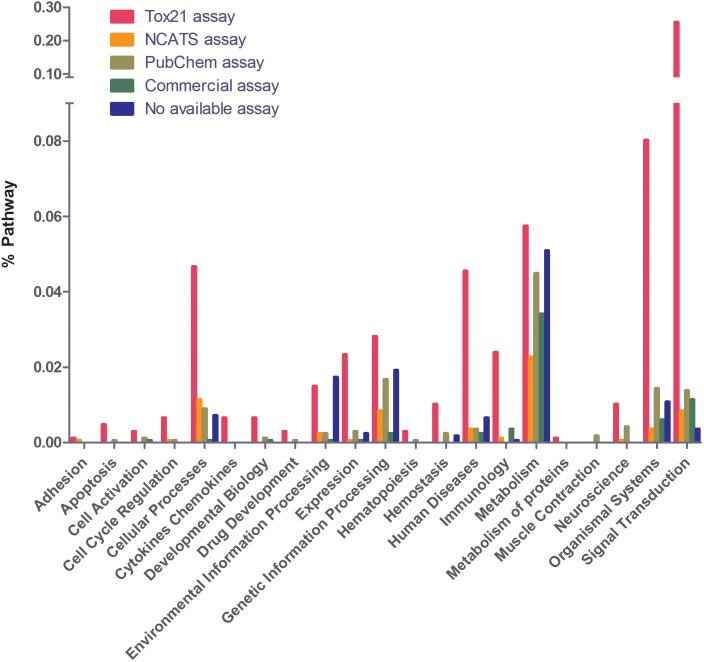
Assay availability for different pathway categories. Cell signaling is the best covered category. Metabolism is the least covered category. Tox21 has the best coverage in human disease pathways.

Next, we examined the assay availability for disease-related and non-related pathways (Figure 3C,D). Of the 1,658 human pathways, 97% contain at least one gene that is implicated in a genetic disease according to OMIM (Figure 3B). As of July 10, 2017, OMIM annotates 15,649 genes, including 6,013 phenotypes (usually diseases) that have been attributed to cognate genes^7^. Genes that cause genetic diseases have been identified in 97% of annotated pathways to date. Disease-related pathways have significantly better assay coverage (89% have at least one bioassay) than pathways that do not contain any disease-related genes (66% have bioassays). Compared to the other PubChem assays, Tox21 and NCATS showed relatively better coverage of disease-related pathways (71% assays are from Tox21 or NCATS) than other pathways (only 40% assays are from Tox21 or NCATS). Figure 4 shows the assay availability for different pathway categories. Cell signaling is by far the best-covered pathway category with 99% of the 488 pathways having a bioassay available. In contrast, metabolism, the second largest pathway category, has only 76% of the 351 pathways having a bioassay from the four assay sources. The human disease pathway category shown in Figure 4 was not defined by having an OMIM gene, but from the annotations obtained from the pathway data sources. Nevertheless, 89% of these 103 pathways identified as human disease pathways have an available bioassay. In fact, 97% of metabolic pathways contain OMIM genes, which is almost the same as the percentage of signaling pathways containing OMIM genes (98%). This suggests that the apparent lack of interest in developing assays to probe metabolic pathways is unwarranted if the drive behind the wide interest in studying signaling pathways is their well acknowledged role in disease processes.

### Probing the Pathway Universe With Minimum Number of Assays

The ultimate goal of systems pharmacology, of which the Tox21 program is an exemplar, is to characterize the activity of a broad range of chemicals across the full spectrum of 1,658 human pathways. However, since performing 1,658 separate assays is experimentally unfeasible, and given that pathways are overlapping in their component genes and functions, and together constitute an interconnected network web, we reasoned that it should be possible to account for all of pathway space with a reduced number of assays that could cover multiple pathways. We thus sought to define a minimal set of gene targets that could be experimentally assayed and cover all of pathway space with some degree of overlap and redundancy to assure complete coverage.

We identified a minimum set of 362 genes that cover the entire list of 1,658 pathways (Supplementary Table S1a). Specifically, genes were first sorted by the number and size of pathways in which they participate, such that genes that appear in more pathways and smaller pathways were ranked higher. An iterative algorithm was then applied to go through the gene list collecting the highest ranked genes while keeping track of the pathways covered by the genes collected. The algorithm stopped when all pathways were covered and the 362 genes collected form the maximum coverage list. As most of these genes participate in multiple pathways, it is not surprising to find that this set of genes is significantly enriched (82 out of 362, *p* < 1.0 × 10^−4^) with genes that have been reported to be essential for the viability of human cells (Blomen et al., 2015; Fraser, 2015). When availability of assays in Tox21, NCATS, PubChem, or commercial sources was taken into account, that is, higher priority was assigned to genes with assays available in one of these three sources, a minimum set of 411 genes was identified to cover all pathways (Supplementary Table S1b). More genes are required in this case because not all the genes that can cover the largest number of pathways have assays available, thus additional genes are needed to cover the same pathways.

The underlying premise of testing compounds in a reduced number of assays as a proxy for all biological pathway activity space is that it is possible to identify “indicator pathways” based on genes that regulate/participate in several pathways, such that activity in this indicator assay would allow inference that the compound would be active in other pathways that share this gene product. In this case, screening multiple pathway assays sharing the same gene target(s) would be redundant and thus unnecessary in a global assessment of compound activity on biological space. This premise predicts a positive correlation between the degree of compound activity overlap and the extent of gene sharing of two pathway assays. To test this prediction, we evaluated data generated from screening of the pilot phase Tox21 collection of 2,870 compounds against a set of 25 pathway assays (Supplementary Table S2). The degrees of gene sharing and activity overlap were calculated for each pathway assay pair. Briefly, the degree of gene sharing between two pathways was defined as the ratio of genes shared by the two pathways over the total number of unique genes in the two pathways. The compound activity overlap between two assays was defined as the ratio of compounds active in both assays over the number of compounds active in either assay. A significant positive correlation was found (*r* = 0.41, *p* < 1.0 × 10^−20^), and the correlation improved to 0.57 when the degree of gene sharing between two pathway assays was >20%. Though this correlation is statistically significant and supports the notion that achievement of a compound’s comprehensive pathway activity footprint via testing in the full 1,658 pathways will be feasible, the extent to which pathway activity may be confidently inferred from activity in other “indicator” assays is unclear and will require experimental testing. One of the major goals of the Tox21 program and other systems biology initiatives is to generate and make public just these kinds of diverse pathway data and predictive algorithms, and experimentally test their utility. As data are generated, they will be linked to the BioPlanet for straightforward browsing and correlation testing by others and ourselves.

### The BioPlanet Pathway Browser

**FIGURE 5:**
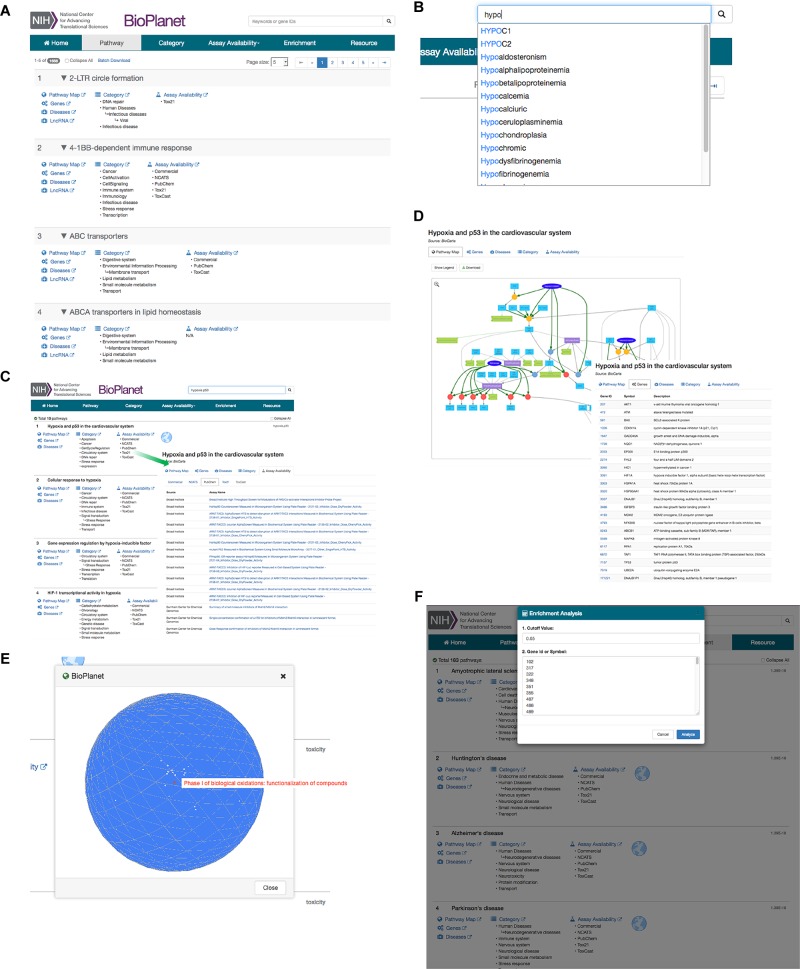
Example use cases of the NCATS BioPlanet web browser (http://tripod.nih.gov/bioplanet). **(A)** Pathway browsing by name, category, or assay availability from main page. **(B)** Free text search with the autosuggest functionality. **(C)** Search results view: a multiple term search example with the keywords “hypoxia” and “p53.” The term(s) used to retrieve each pathway, “hypoxia” and/or “p53,” is shown on the right. The pathway title, when clicked on, expands to show detailed annotations such as assay availability, category, disease relevance, with links to outside sources when available. This example shows the assays available in PubChem for the first pathway retrieved. **(D)** Pathway detail view with an interactive pathway diagram and its gene component list. **(E)** The 3-dimensional globe view that shows a group of pathways projected on the globe. Each dot on the globe represents a pathway. Mousing over the pathway shows the pathway name. **(F)** Enrichment analysis tool that allows users to paste in a list of genes and determine which BioPlanet pathways are enriched in the gene list. The significance of enrichment *p*-value is shown on the right of each pathway returned.

We report here what we believe to be the most comprehensive non-redundant enumeration to date of pathways extant in human cells, and the connections between them. To facilitate the browsing, visualization, and analysis of the pathway universe, we have constructed a unified database and a web-based software platform, the NCATS BioPlanet^8^, that is publicly available (Figure 5). From the main page, users may browse pathways by name, category, or assay availability (Figure 5A). The BioPlanet web browser also supports free text search enhanced by the availability of autocomplete suggestions as shown in Figure 5B. Users may search the BioPlanet by keywords, such as those that appear in a gene or pathway name, or a disease, or gene identifiers such as Entrez gene IDs. Batch search is also supported, that allows a user to paste in multiple gene IDs or keywords and retrieve their records via a single query. Search is performed on each individual search term as well as combinations of terms, and each pathway returned is labeled by the searched term(s) used to retrieve that pathway (Figure 5C). In the search results view, each pathway is labeled with functional category tags, disease relevance, and assay availability (Figure 5C). References to the original data sources are also provided. Each search result is a card that contains links to all pathway details: Pathway Map, Genes, ncRNAs, Diseases, Categories, and Assays.

In particular, Pathway Map is the most detailed graphical representation of a pathway demonstrating all known interactions between genes, proteins, nucleic acids, and small molecules in that pathway (Figure 5D). Importantly, these maps show the entirety of the pathway data stored in BioPAX or SBML formats obtained from public sources (vide supra), and curated, and thus provide the highest amount of detail known to date, without compromising the visual clarity. Moreover, this pathway diagram is searchable and interactive, where a click on each component will show a tooltip with known literature references and identifiers.

The browser also provides the mapping of pathways on a 3-dimensional globe, in which the distance between any two pathways on the globe surface is proportional to their degree of their gene component overlap (Figure 5E). This allows users to conduct a pathway similarity analysis at a glance, and demonstrates the interaction between different biological processes.

A gene enrichment analysis tool is also provided where the user can input a list of genes and determine which BioPlanet pathways are enriched in said list (Figure 5F).

## Discussion

The Human Genome Project ushered in a continuing era of comprehensive enumeration of all biological system components. Building on human and model organism reference genome sequences, comprehensive identification or production of genes (Collins et al., 1998, 2003), cDNAs (Strausberg et al., 1999; Gerhard et al., 2004; Temple et al., 2009), SNPs and haplotypes (International HapMap Consortium, 2003; Thorisson et al., 2005), structural and functional elements of genomes (ENCODE Project Consortium, 2004; Birney et al., 2007; Celniker et al., 2009), knockout mice (Austin et al., 2004), transcriptomes (Katayama et al., 2005), and microRNAs (He and Hannon, 2004; Bentwich et al., 2005) have been accomplished. Excellent efforts at enumeration of molecular, metabolic, and signaling pathways have been undertaken by multiple groups, but to date there has not been a synthesis of these efforts into a single collection of all pathways operant in human cells. The BioPlanet is the first attempt at creating such a resource, aiming to be comprehensive, non-redundant, relational, and easy to navigate.

Furthermore, the BioPlanet pathways are extensively annotated in terms of functional categories, disease relevance, assay availability and lncRNA regulation, which seems to be insufficient or lacking in various pathway databases. Using disease pathways as an example, most data sources we examined do not have explicit indications on which pathways have been associated with diseases. BioCarta and Reactome sort their pathways into several different categories but a general category for disease pathways is not available. KEGG is the only database with a “human diseases” category, but the genes listed in these human disease pathways only account for 27% of the OMIM disease genes. This shows that many pathways that might have disease relevance have not been explicitly annotated as such in previous pathway databases. Since one of our aims in creating the NCATS BioPlanet database was to enumerate a complete and non-redundant listing of all human disease-related pathways, we included the prevalence of disease genes as a principal feature in annotating all pathways in the BioPlanet. In addition, we manually examined and assigned a category to each pathway that did not have a category annotation in its source database. Furthermore, the complete and non-redundant feature of the BioPlanet would enable users to not only get a complete and concise interpretation of their experimental results from, e.g., genomic or proteomic screens, but also design an optimal set of targets or *in vitro* assays to comprehensively interrogate the biological space as detailed later below. This would not be possible with any other existing databases.

It is important to emphasize that BioPlanet, like other cataloging efforts before it, is an attempt to represent complex and often state-dependent systems in a uniform way and as such is subject to oversimplification. In addition, since the BioPlanet is built on a foundation of current understanding of pathways and their interconnections, there are undoubtedly errors in it, both representational and biological. We, therefore, view the BioPlanet as a work in progress, and designate the version currently available on our website (see text footnote 8) as BioPlanet 1.0 in recognition of its evolving nature. Like the data that went into creating the current version of BioPlanet, which was derived from the community of scientists worldwide, we view the ongoing curation and growth of the BioPlanet as a community “wiki” type effort, and therefore actively encourage comments, corrections, contributions, and suggestions for additional features via the BioPlanet page at http://tripod.nih.gov/bioplanet. All contributions will be acknowledged and attributed on this page.

We hope that the research community will find the BioPlanet useful, both for systems biology analyses as well as hypothesis generation. However, beyond its utility as a catalog, we hope that the BioPlanet will facilitate perturbation studies using small molecules, RNAi, gene knockouts, and other forms of biological modulation. The ultimate test of any network map is its ability to predict effects when a node in the network is perturbed. We will be adding capabilities for linking data from small molecule and siRNA screens performed at our Center to the next version of the BioPlanet, and we look forward to linking data obtained by other researchers as well. The current version of BioPlanet contains only human pathways, therefore, as a future endeavor, pathways for other species will be added both for their own importance in biological research and in comparison to their human counterparts, since human-animal pathway differences are likely drivers of non-concordance of chemical effects on humans and animals.

In the nearest term, the BioPlanet will find utility in the selection of *in vitro* assays to strengthen predictive toxicology methods (National Research Council [NRC], 2007; Kavlock et al., 2009). An underlying premise of *in vitro* toxicology approaches is that any pathway which plays an important role in human physiology could, if sufficiently perturbed, yield pathophysiology, i.e., toxicity. As we have demonstrated recently, an optimally designed panel of *in vitro* assays with targets diverse enough to sufficiently cover the biological response space could achieve good performance in predicting *in vivo* human toxicity, such as adverse drug effects (Huang et al., 2016, 2018). The BioPlanet would be an ideal guiding tool in designing such an assay panel. By analogy to genome-wide association studies (GWAS), we might refer to the present *in vitro* toxicology approaches, such as Tox21, as “pathway-ome-wide activity study,” or PWAS. Like GWAS studies, in which querying of all polymorphisms in the genomes of thousands of participants has been considered impractical, PWAS of all 1,658 pathways across thousands of chemicals is similarly difficult: by way of example, a 15-point concentration-response quantitative high throughput (qHTS) screen of the Tox21 “10K” set requires at least one week for each assay even with the ultrahigh-throughput robotic platform being utilized. GWAS studies were rendered practical by the comprehensive cataloging of SNPs and the discovery of the SNPs that are inherited together in haplotype blocks, thus allowing the imputation of SNPs not directly tested via the presence of a reduced numbers of “tag SNPs.” While there are 1,658 total pathways currently characterized, our analysis suggests that assaying only 362 will allow the imputation of activity in the remaining pathways. Importantly, this reduced number, while consistent with current data, will require ongoing data production to test and refine this very concept and the actual number of independent assays required to adequately query all of pathway activity space, with such data being provided in PubChem and other public-facing portals on a continuing basis.

While BioPlanet was initially conceived as a tool to guide systems toxicology efforts, it has implications and applications across the spectrum of systems biology, systems pharmacology, and disease pathophysiology. We look forward to continuing to collaborate with the research community to further develop and populate the BioPlanet, and thus achieve its potential as a resource for discovery.

## Author Contributions

RH coordinated the project, sourced and compiled pathway lists to construct the BioPlanet database, helped with data curation, helped to build the BioPlanet database and browser, performed statistical analysis of all data, and wrote the manuscript. TZ and YW built the BioPlanet database and browser. IG, JG, and JO curated all data and wrote the manuscript. IG generated all pathway diagrams. DN helped with assay vender searching and data curation. D-TN helped to extract data from PubChem and build the BioPlanet browser. RG helped with data curation and the BioPlanet browser. AJ coordinated the data curation. NS helped to coordinate data curation and construction of the BioPlanet browser. AS directed the project and wrote the manuscript. CA conceived and directed the project and wrote the manuscript. All authors reviewed the manuscript.

## Conflict of Interest Statement

IG, JG, and JO were employed by company Rancho BioSciences. The remaining authors declare that the research was conducted in the absence of any commercial or financial relationships that could be construed as a potential conflict of interest.

## Footnotes

1 http://humancyc.org/

2 http://david.abcc.ncifcrf.gov

3 http://www.ncbi.nlm.nih.gov/gene/about-generif

4 http://geneontology.org/docs/go-subset-guide/

5 http://disease-ontology.org/

6 http://ctdbase.org/

7 http://omim.org/statistics/entry

8 http://tripod.nih.gov/bioplanet
