# Supplementary material for: The NCATS BioPlanet – An Integrated Platform for Exploring the Universe of Cellular Signaling Pathways for Toxicology, Systems Biology, and Chemical Genomics
Source: Front Pharmacol. 2019 Apr 26;10:445. doi: 10.3389/fphar.2019.00445 (PMC6524730; doi:10.3389/fphar.2019.00445)
Supplement: Supplementary file 1 [file Data_Sheet_1.pdf]

Table S1. Genes that cover the entire list of 1,658 pathways (a) regardless of assay availability, and (b) with probing assays available

(a)

Entrez Gene ID	Gene	Description
5595	MAPK3	mitogen-activated protein kinase 3
5594	MAPK1	mitogen-activated protein kinase 1
207	AKT1	v-akt murine thymoma viral oncogene homolog 1
5604	MAP2K1	mitogen-activated protein kinase kinase 1
5295	PIK3R1	phosphoinositide-3-kinase, regulatory subunit 1 (alpha)
5290	PIK3CA	phosphoinositide-3-kinase, catalytic, alpha polypeptide
3265	HRAS	v-Ha-ras Harvey rat sarcoma viral oncogene homolog
5599	MAPK8	mitogen-activated protein kinase 8
6714	SRC	v-src sarcoma (Schmidt-Ruppin A-2) viral oncogene homolog (avian)
5578	PRKCA	protein kinase C, alpha
2885	GRB2	growth factor receptor-bound protein 2
983	CDK1	cyclin-dependent kinase 1
4790	NFKB1	nuclear factor of kappa light polypeptide gene enhancer in B-cells 1
5894	RAF1	v-raf-1 murine leukemia viral oncogene homolog 1
5879	RAC1	ras-related C3 botulinum toxin substrate 1 (rho family, small GTP binding protein Rac1)
1432	MAPK14	mitogen-activated protein kinase 14
5970	RELA	v-rel reticuloendotheliosis viral oncogene homolog A (avian)
6654	SOS1	son of sevenless homolog 1 (Drosophila)
5605	MAP2K2	mitogen-activated protein kinase kinase 2
387	RHOA	ras homolog gene family, member A
1385	CREB1	cAMP responsive element binding protein 1
6464	SHC1	SHC (Src homology 2 domain containing) transforming protein 1
5170	PDPK1	3-phosphoinositide dependent protein kinase-1
3725	JUN	jun proto-oncogene
208	AKT2	v-akt murine thymoma viral oncogene homolog 2
5567	PRKACB	protein kinase, cAMP-dependent, catalytic, beta
1147	CHUK	conserved helix-loop-helix ubiquitous kinase
1387	CREBBP	CREB binding protein
5335	PLCG1	phospholipase C, gamma 1
2353	FOS	FBJ murine osteosarcoma viral oncogene homolog
2534	FYN	FYN oncogene related to SRC, FGR, YES
1956	EGFR	epidermal growth factor receptor
801	CALM1	calmodulin 1 (phosphorylase kinase, delta)
3688	ITGB1	integrin, beta 1 (fibronectin receptor, beta polypeptide, antigen CD29 includes MDF2, MSK12)

7124	TNF	tumor necrosis factor
920	CD4	CD4 molecule
107	ADCY1	adenylate cyclase 1 (brain)
4803	NGF	nerve growth factor (beta polypeptide)
836	CASP3	caspase 3, apoptosis-related cysteine peptidase
7040	TGFB1	transforming growth factor, beta 1
6774	STAT3	signal transducer and activator of transcription 3 (acute-phase response factor)
5566	PRKACA	protein kinase, cAMP-dependent, catalytic, alpha
7157	TP53	tumor protein p53
596	BCL2	B-cell CLL/lymphoma 2
2782	GNB1	guanine nucleotide binding protein (G protein), beta polypeptide 1
1869	E2F1	E2F transcription factor 1
5781	PTPN11	protein tyrosine phosphatase, non-receptor type 11
5747	PTK2	PTK2 protein tyrosine kinase 2
7316	UBC	ubiquitin C
6233	RPS27A	ribosomal protein S27a
7311	UBA52	ubiquitin A-52 residue ribosomal protein fusion product 1
3932	LCK	lymphocyte-specific protein tyrosine kinase
842	CASP9	caspase 9, apoptosis-related cysteine peptidase
6256	RXRA	retinoid X receptor, alpha
2932	GSK3B	glycogen synthase kinase 3 beta
841	CASP8	caspase 8, apoptosis-related cysteine peptidase
5925	RB1	retinoblastoma 1
23236	PLCB1	phospholipase C, beta 1 (phosphoinositide-specific)
5515	PPP2CA	protein phosphatase 2, catalytic subunit, alpha isozyme
7189	TRAF6	TNF receptor-associated factor 6
995	CDC25C	cell division cycle 25 homolog C (S. pombe)
3320	HSP90AA1	heat shock protein 90kDa alpha (cytosolic), class A member 1
2475	MTOR	mechanistic target of rapamycin (serine/threonine kinase)
1017	CDK2	cyclin-dependent kinase 2
3558	IL2	interleukin 2
4609	MYC	v-myc myelocytomatosis viral oncogene homolog (avian)
1499	CTNNB1	catenin (cadherin-associated protein), beta 1, 88kDa
6772	STAT1	signal transducer and activator of transcription 1, 91kDa
598	BCL2L1	BCL2-like 1
355	FAS	Fas (TNF receptor superfamily, member 6)
472	ATM	ataxia telangiectasia mutated
3576	IL8	interleukin 8
3065	HDAC1	histone deacetylase 1
7422	VEGFA	vascular endothelial growth factor A
999	CDH1	cadherin 1, type 1, E-cadherin (epithelial)

3458	IFNG	interferon, gamma
3630	INS	insulin
6117	RPA1	replication protein A1, 70kDa
4851	NOTCH1	notch 1
5321	PLA2G4A	phospholipase A2, group IVA (cytosolic, calcium-dependent)
595	CCND1	cyclin D1
3030	HADHA	hydroxyacyl-CoA dehydrogenase/3-ketoacyl-CoA thiolase/enoyl-CoA hydratase (trifunctional protein), alpha subunit
4846	NOS3	nitric oxide synthase 3 (endothelial cell)
3569	IL6	interleukin 6 (interferon, beta 2)
2147	F2	coagulation factor II (thrombin)
4128	MAOA	monoamine oxidase A
919	CD247	CD247 molecule
5829	PXN	paxillin
3553	IL1B	interleukin 1, beta
5580	PRKCD	protein kinase C, delta
2002	ELK1	ELK1, member of ETS oncogene family
1173	AP2M1	adaptor-related protein complex 2, mu 1 subunit
54205	CYCS	cytochrome c, somatic
38	ACAT1	acetyl-CoA acetyltransferase 1
7504	XK	X-linked Kx blood group (McLeod syndrome)
5347	PLK1	polo-like kinase 1
834	CASP1	caspase 1, apoptosis-related cysteine peptidase (interleukin 1, beta, convertase)
5663	PSEN1	presenilin 1
5045	FURIN	furin (paired basic amino acid cleaving enzyme)
2246	FGF1	fibroblast growth factor 1 (acidic)
2805	GOT1	glutamic-oxaloacetic transaminase 1, soluble (aspartate aminotransferase 1)
5516	PPP2CB	protein phosphatase 2, catalytic subunit, beta isozyme
1892	ECHS1	enoyl CoA hydratase, short chain, 1, mitochondrial
1022	CDK7	cyclin-dependent kinase 7
3383	ICAM1	intercellular adhesion molecule 1
2697	GJA1	gap junction protein, alpha 1, 43kDa
8074	FGF23	fibroblast growth factor 23
718	C3	complement component 3
1977	EIF4E	eukaryotic translation initiation factor 4E
1576	CYP3A4	cytochrome P450, family 3, subfamily A, polypeptide 4
4089	SMAD4	SMAD family member 4
816	CAMK2B	calcium/calmodulin-dependent protein kinase II beta
58	ACTA1	actin, alpha 1, skeletal muscle
1544	CYP1A2	cytochrome P450, family 1, subfamily A, polypeptide 2
3122	HLA-DRA	major histocompatibility complex, class II, DR alpha

293	SLC25A6	solute carrier family 25 (mitochondrial carrier; adenine nucleotide translocator), member 6
3091	HIF1A	hypoxia inducible factor 1, alpha subunit (basic helix-loop-helix transcription factor)
5581	PRKCE	protein kinase C, epsilon
135	ADORA2A	adenosine A2a receptor
839	CASP6	caspase 6, apoptosis-related cysteine peptidase
2547	XRCC6	X-ray repair complementing defective repair in Chinese hamster cells 6
8815	BANF1	barrier to autointegration factor 1
7249	TSC2	tuberous sclerosis 2
6470	SHMT1	serine hydroxymethyltransferase 1 (soluble)
6898	TAT	tyrosine aminotransferase
672	BRCA1	breast cancer 1, early onset
6722	SRF	serum response factor (c-fos serum response element-binding transcription factor)
351	APP	amyloid beta (A4) precursor protein
1081	CGA	glycoprotein hormones, alpha polypeptide
5111	PCNA	proliferating cell nuclear antigen
5155	PDGFB	platelet-derived growth factor beta polypeptide (simian sarcoma viral (v-sis) oncogene homolog)
3685	ITGAV	integrin, alpha V (vitronectin receptor, alpha polypeptide, antigen CD51)
3295	HSD17B4	hydroxysteroid (17-beta) dehydrogenase 4
3014	H2AFX	H2A histone family, member X
5563	PRKAA2	protein kinase, AMP-activated, alpha 2 catalytic subunit
6582	SLC22A2	solute carrier family 22 (organic cation transporter), member 2
1020	CDK5	cyclin-dependent kinase 5
5610	EIF2AK2	eukaryotic translation initiation factor 2-alpha kinase 2
1211	CLTA	clathrin, light chain A
6844	VAMP2	vesicle-associated membrane protein 2 (synaptobrevin 2)
1965	EIF2S1	eukaryotic translation initiation factor 2, subunit 1 alpha, 35kDa
7450	VWF	von Willebrand factor
7514	XPO1	exportin 1 (CRM1 homolog, yeast)
5743	PTGS2	prostaglandin-endoperoxide synthase 2 (prostaglandin G/H synthase and cyclooxygenase)
501	ALDH7A1	aldehyde dehydrogenase 7 family, member A1
3479	IGF1	insulin-like growth factor 1 (somatomedin C)
3708	ITPR1	inositol 1,4,5-triphosphate receptor, type 1
5328	PLAU	plasminogen activator, urokinase
2966	GTF2H2	general transcription factor IIH, polypeptide 2, 44kDa
8106	PABPN1	poly(A) binding protein, nuclear 1
2328	FMO3	flavin containing monooxygenase 3
2255	FGF10	fibroblast growth factor 10

9965	FGF19	fibroblast growth factor 19
1593	CYP27A1	cytochrome P450, family 27, subfamily A, polypeptide 1
1644	DDC	dopa decarboxylase (aromatic L-amino acid decarboxylase)
1374	CPT1A	carnitine palmitoyltransferase 1A (liver)
4023	LPL	lipoprotein lipase
54658	UGT1A1	UDP glucuronosyltransferase 1 family, polypeptide A1
8818	DPM2	dolichyl-phosphate mannosyltransferase polypeptide 2, regulatory subunit
2064	ERBB2	v-erb-b2 erythroblastic leukemia viral oncogene homolog 2, neuro/glioblastoma derived oncogene homolog (avian)
3949	LDLR	low density lipoprotein receptor
55902	ACSS2	acyl-CoA synthetase short-chain family member 2
2891	GRIA2	glutamate receptor, ionotropic, AMPA 2
2159	F10	coagulation factor X
5337	PLD1	phospholipase D1, phosphatidylcholine-specific
3661	IRF3	interferon regulatory factor 3
11343	MGLL	monoglyceride lipase
727	C5	complement component 5
4121	MAN1A1	mannosidase, alpha, class 1A, member 1
51085	MLXIPL	MLX interacting protein-like
4998	ORC1	origin recognition complex, subunit 1
5243	ABCB1	ATP-binding cassette, sub-family B (MDR/TAP), member 1
2784	GNB3	guanine nucleotide binding protein (G protein), beta polypeptide 3
51606	ATP6V1H	ATPase, H+ transporting, lysosomal 50/57kDa, V1 subunit H
7360	UGP2	UDP-glucose pyrophosphorylase 2
1128	CHRM1	cholinergic receptor, muscarinic 1
5901	RAN	RAN, member RAS oncogene family
5053	PAH	phenylalanine hydroxylase
1571	CYP2E1	cytochrome P450, family 2, subfamily E, polypeptide 1
5422	POLA1	polymerase (DNA directed), alpha 1, catalytic subunit
2626	GATA4	GATA binding protein 4
6916	TBXAS1	thromboxane A synthase 1 (platelet)
6513	SLC2A1	solute carrier family 2 (facilitated glucose transporter), member 1
7341	SUMO1	SMT3 suppressor of mif two 3 homolog 1 (S. cerevisiae)
7321	UBE2D1	ubiquitin-conjugating enzyme E2D 1 (UBC4/5 homolog, yeast)
5440	POLR2K	polymerase (RNA) II (DNA directed) polypeptide K, 7.0kDa
3105	HLA-A	major histocompatibility complex, class I, A
5443	POMC	proopiomelanocortin
5329	PLAUR	plasminogen activator, urokinase receptor
5903	RANBP2	RAN binding protein 2
375	ARF1	ADP-ribosylation factor 1
25	ABL1	c-abl oncogene 1, non-receptor tyrosine kinase
3073	HEXA	hexosaminidase A (alpha polypeptide)

1119	CHKA	choline kinase alpha
3283	HSD3B1	hydroxy-delta-5-steroid dehydrogenase, 3 beta- and steroid delta-isomerase 1
6091	ROBO1	roundabout, axon guidance receptor, homolog 1 (Drosophila)
11001	SLC27A2	solute carrier family 27 (fatty acid transporter), member 2
5430	POLR2A	polymerase (RNA) II (DNA directed) polypeptide A, 220kDa
811	CALR	calreticulin
6915	TBXA2R	thromboxane A2 receptor
3655	ITGA6	integrin, alpha 6
9150	CTDP1	CTD (carboxy-terminal domain, RNA polymerase II, polypeptide A) phosphatase, subunit 1
60	ACTB	actin, beta
1719	DHFR	dihydrofolate reductase
1282	COL4A1	collagen, type IV, alpha 1
231	AKR1B1	aldo-keto reductase family 1, member B1 (aldose reductase)
10249	GLYAT	glycine-N-acyltransferase
1742	DLG4	discs, large homolog 4 (Drosophila)
2645	GCK	glucokinase (hexokinase 4)
1733	DIO1	deiodinase, iodothyronine, type I
1634	DCN	decorin
124	ADH1A	alcohol dehydrogenase 1A (class I), alpha polypeptide
1610	DAO	D-amino-acid oxidase
29968	PSAT1	phosphoserine aminotransferase 1
2817	GPC1	glypican 1
51166	AADAT	aminoadipate aminotransferase
150	ADRA2A	adrenergic, alpha-2A-, receptor
4190	MDH1	malate dehydrogenase 1, NAD (soluble)
1468	SLC25A10	solute carrier family 25 (mitochondrial carrier; dicarboxylate transporter), member 10
8714	ABCC3	ATP-binding cassette, sub-family C (CFTR/MRP), member 3
1594	CYP27B1	cytochrome P450, family 27, subfamily B, polypeptide 1
5167	ENPP1	ectonucleotide pyrophosphatase/phosphodiesterase 1
103	ADAR	adenosine deaminase, RNA-specific
6720	SREBF1	sterol regulatory element binding transcription factor 1
19	ABCA1	ATP-binding cassette, sub-family A (ABC1), member 1
570	BAAT	bile acid CoA: amino acid N-acyltransferase (glycine N-choloyltransferase)
5160	PDHA1	pyruvate dehydrogenase (lipoamide) alpha 1
7498	XDH	xanthine dehydrogenase
4686	NCBP1	nuclear cap binding protein subunit 1, 80kDa
55300	PI4K2B	phosphatidylinositol 4-kinase type 2 beta
2744	GLS	glutaminase
23169	SLC35D1	solute carrier family 35 (UDP-glucuronic acid/UDP-N-acetylgalactosamine dual transporter), member D1

6569	SLC34A1	solute carrier family 34 (sodium phosphate), member 1
4057	LTF	lactotransferrin
6507	SLC1A3	solute carrier family 1 (glial high affinity glutamate transporter), member 3
1573	CYP2J2	cytochrome P450, family 2, subfamily J, polypeptide 2
2678	GGT1	gamma-glutamyltransferase 1
3596	IL13	interleukin 13
7015	TERT	telomerase reverse transcriptase
64240	ABCG5	ATP-binding cassette, sub-family G (WHITE), member 5
2194	FASN	fatty acid synthase
1136	CHRNA3	cholinergic receptor, nicotinic, alpha 3
4968	OGG1	8-oxoguanine DNA glycosylase
3170	FOXA2	forkhead box A2
286	ANK1	ankyrin 1, erythrocytic
6817	SULT1A1	sulfotransferase family, cytosolic, 1A, phenol-preferring, member 1
2864	FFAR1	free fatty acid receptor 1
5584	PRKCI	protein kinase C, iota
6005	RHAG	Rh-associated glycoprotein
6539	SLC6A12	solute carrier family 6 (neurotransmitter transporter, betaine/GABA), member 12
2746	GLUD1	glutamate dehydrogenase 1
183	AGT	angiotensinogen (serpin peptidase inhibitor, clade A, member 8)
4122	MAN2A2	mannosidase, alpha, class 2A, member 2
8884	SLC5A6	solute carrier family 5 (sodium-dependent vitamin transporter), member 6
3356	HTR2A	5-hydroxytryptamine (serotonin) receptor 2A
775	CACNA1C	calcium channel, voltage-dependent, L type, alpha 1C subunit
213	ALB	albumin
5406	PNLIP	pancreatic lipase
6482	ST3GAL1	ST3 beta-galactoside alpha-2,3-sialyltransferase 1
23539	SLC16A8	solute carrier family 16, member 8 (monocarboxylic acid transporter 3)
10057	ABCC5	ATP-binding cassette, sub-family C (CFTR/MRP), member 5
6790	AURKA	aurora kinase A
3776	KCNK2	potassium channel, subfamily K, member 2
551	AVP	arginine vasopressin
84515	MCM8	minichromosome maintenance complex component 8
229	ALDOB	aldolase B, fructose-bisphosphate
50674	NEUROG3	neurogenin 3
2987	GUK1	guanylate kinase 1
488	ATP2A2	ATPase, Ca++ transporting, cardiac muscle, slow twitch 2
7098	TLR3	toll-like receptor 3
412	STS	steroid sulfatase (microsomal), isozyme S
2569	GABRR1	gamma-aminobutyric acid (GABA) receptor, rho 1
8443	GNPAT	glyceronephosphate O-acyltransferase

8898	MTMR2	myotubularin related protein 2
3766	KCNJ10	potassium inwardly-rectifying channel, subfamily J, member 10
2673	GFPT1	glutamine--fructose-6-phosphate transaminase 1
7351	UCP2	uncoupling protein 2 (mitochondrial, proton carrier)
6548	SLC9A1	solute carrier family 9 (sodium/hydrogen exchanger), member 1
7037	TFRC	transferrin receptor (p90, CD71)
6996	TDG	thymine-DNA glycosylase
10800	CYSLTR1	cysteinyl leukotriene receptor 1
1813	DRD2	dopamine receptor D2
376497	SLC27A1	solute carrier family 27 (fatty acid transporter), member 1
2582	GALE	UDP-galactose-4-epimerase
1915	EEF1A1	eukaryotic translation elongation factor 1 alpha 1
27010	TPK1	thiamin pyrophosphokinase 1
3417	IDH1	isocitrate dehydrogenase 1 (NADP+), soluble
161823	ADAL	adenosine deaminase-like
10459	MAD2L2	MAD2 mitotic arrest deficient-like 2 (yeast)
2683	B4GALT1	UDP-Gal:betaGlcNAc beta 1,4- galactosyltransferase, polypeptide 1
8321	FZD1	frizzled homolog 1 (Drosophila)
6518	SLC2A5	solute carrier family 2 (facilitated glucose/fructose transporter), member 5
1158	CKM	creatine kinase, muscle
3039	HBA1	hemoglobin, alpha 1
7319	UBE2A	ubiquitin-conjugating enzyme E2A (RAD6 homolog)
27161	EIF2C2	eukaryotic translation initiation factor 2C, 2
6523	SLC5A1	solute carrier family 5 (sodium/glucose cotransporter), member 1
3309	HSPA5	heat shock 70kDa protein 5 (glucose-regulated protein, 78kDa)
4594	MUT	methylmalonyl CoA mutase
64902	AGXT2	alanine--glyoxylate aminotransferase 2
60490	PPCDC	phosphopantothenoylcysteine decarboxylase
6833	ABCC8	ATP-binding cassette, sub-family C (CFTR/MRP), member 8
1901	S1PR1	sphingosine-1-phosphate receptor 1
4351	MPI	mannose phosphate isomerase
760	CA2	carbonic anhydrase II
7298	TYMS	thymidylate synthetase
9695	EDEM1	ER degradation enhancer, mannosidase alpha-like 1
92	ACVR2A	activin A receptor, type IIA
114134	SLC2A13	solute carrier family 2 (facilitated glucose transporter), member 13
211	ALAS1	aminolevulinate, delta-, synthase 1
6546	SLC8A1	solute carrier family 8 (sodium/calcium exchanger), member 1
7019	TFAM	transcription factor A, mitochondrial
2554	GABRA1	gamma-aminobutyric acid (GABA) A receptor, alpha 1
4255	MGMT	O-6-methylguanine-DNA methyltransferase

51128	SAR1B	SAR1 homolog B (S. cerevisiae)
64816	CYP3A43	cytochrome P450, family 3, subfamily A, polypeptide 43
11019	LIAS	lipoic acid synthetase
3759	KCNJ2	potassium inwardly-rectifying channel, subfamily J, member 2
10585	POMT1	protein-O-mannosyltransferase 1
3067	HDC	histidine decarboxylase
11255	HRH3	histamine receptor H3
57369	GJD2	gap junction protein, delta 2, 36kDa
6628	SNRPB	small nuclear ribonucleoprotein polypeptides B and B1
9054	NFS1	NFS1 nitrogen fixation 1 homolog (S. cerevisiae)
10914	PAPOLA	poly(A) polymerase alpha
5393	EXOSC9	exosome component 9
284111	SLC13A5	solute carrier family 13 (sodium-dependent citrate transporter), member 5
435	ASL	argininosuccinate lyase
8877	SPHK1	sphingosine kinase 1
5819	PVRL2	poliovirus receptor-related 2 (herpesvirus entry mediator B)
148867	SLC30A7	solute carrier family 30 (zinc transporter), member 7
1890	TYMP	thymidine phosphorylase
6521	SLC4A1	solute carrier family 4, anion exchanger, member 1 (erythrocyte membrane protein band 3, Diego blood group)
6010	RHO	rhodopsin
121260	SLC15A4	solute carrier family 15, member 4
167227	DCP2	DCP2 decapping enzyme homolog (S. cerevisiae)
8824	CES2	carboxylesterase 2
358	AQP1	aquaporin 1 (Colton blood group)
610	HCN2	hyperpolarization activated cyclic nucleotide-gated potassium channel 2
11194	ABCB8	ATP-binding cassette, sub-family B (MDR/TAP), member 8
7277	TUBA4A	tubulin, alpha 4a
3375	IAPP	islet amyloid polypeptide
498	ATP5A1	ATP synthase, H+ transporting, mitochondrial F1 complex, alpha subunit 1, cardiac muscle
10135	NAMPT	nicotinamide phosphoribosyltransferase
784	CACNB3	calcium channel, voltage-dependent, beta 3 subunit
50515	CHST11	carbohydrate (chondroitin 4) sulfotransferase 11
27173	SLC39A1	solute carrier family 39 (zinc transporter), member 1
6865	TACR2	tachykinin receptor 2
29127	RACGAP1	Rac GTPase activating protein 1
2030	SLC29A1	solute carrier family 29 (nucleoside transporters), member 1
3778	KCNMA1	potassium large conductance calcium-activated channel, subfamily M, alpha member 1
7965	AIMP2	aminoacyl tRNA synthetase complex-interacting multifunctional protein 2
6558	SLC12A2	solute carrier family 12 (sodium/potassium/chloride transporters), member 2

2911	GRM1	glutamate receptor, metabotropic 1
3737	KCNA2	potassium voltage-gated channel, shaker-related subfamily, member 2
8972	MGAM	maltase-glucoamylase (alpha-glucosidase)
5471	PPAT	phosphoribosyl pyrophosphate amidotransferase
11285	B4GALT7	xylosylprotein beta 1,4-galactosyltransferase, polypeptide 7 (galactosyltransferase I)
115111	SLC26A7	solute carrier family 26, member 7
59350	RXFP1	relaxin/insulin-like family peptide receptor 1
55699	IARS2	isoleucyl-tRNA synthetase 2, mitochondrial
3061	HCRTR1	hypocretin (orexin) receptor 1

(b)

**Entrez Gene ID**	**Gene**	**Description**
5595	MAPK3	mitogen-activated protein kinase 3
5594	MAPK1	mitogen-activated protein kinase 1
207	AKT1	v-akt murine thymoma viral oncogene homolog 1
5604	MAP2K1	mitogen-activated protein kinase kinase 1
5290	PIK3CA	phosphoinositide-3-kinase, catalytic, alpha polypeptide
3265	HRAS	v-Ha-ras Harvey rat sarcoma viral oncogene homolog
5599	MAPK8	mitogen-activated protein kinase 8
6714	SRC	v-src sarcoma (Schmidt-Ruppin A-2) viral oncogene homolog (avian)
5578	PRKCA	protein kinase C, alpha
983	CDK1	cyclin-dependent kinase 1
4790	NFKB1	nuclear factor of kappa light polypeptide gene enhancer in B-cells 1
5894	RAF1	v-raf-1 murine leukemia viral oncogene homolog 1
5879	RAC1	ras-related C3 botulinum toxin substrate 1 (rho family, small GTP binding protein Rac1)
1432	MAPK14	mitogen-activated protein kinase 14
5970	RELA	v-rel reticuloendotheliosis viral oncogene homolog A (avian)
5605	MAP2K2	mitogen-activated protein kinase kinase 2
1385	CREB1	cAMP responsive element binding protein 1
5170	PDPK1	3-phosphoinositide dependent protein kinase-1
3725	JUN	jun proto-oncogene
208	AKT2	v-akt murine thymoma viral oncogene homolog 2
5567	PRKACB	protein kinase, cAMP-dependent, catalytic, beta
1147	CHUK	conserved helix-loop-helix ubiquitous kinase
1387	CREBBP	CREB binding protein
5335	PLCG1	phospholipase C, gamma 1
2353	FOS	FBJ murine osteosarcoma viral oncogene homolog
2534	FYN	FYN oncogene related to SRC, FGR, YES

1956	EGFR	epidermal growth factor receptor
3688	ITGB1	integrin, beta 1 (fibronectin receptor, beta polypeptide, antigen CD29 includes MDF2, MSK12)
7124	TNF	tumor necrosis factor
4803	NGF	nerve growth factor (beta polypeptide)
836	CASP3	caspase 3, apoptosis-related cysteine peptidase
7040	TGFB1	transforming growth factor, beta 1
6774	STAT3	signal transducer and activator of transcription 3 (acute-phase response factor)
5566	PRKACA	protein kinase, cAMP-dependent, catalytic, alpha
7157	TP53	tumor protein p53
1869	E2F1	E2F transcription factor 1
8517	IKBKG	inhibitor of kappa light polypeptide gene enhancer in B-cells, kinase gamma
5781	PTPN11	protein tyrosine phosphatase, non-receptor type 11
5747	PTK2	PTK2 protein tyrosine kinase 2
6233	RPS27A	ribosomal protein S27a
7314	UBB	ubiquitin B
3932	LCK	lymphocyte-specific protein tyrosine kinase
842	CASP9	caspase 9, apoptosis-related cysteine peptidase
6256	RXRA	retinoid X receptor, alpha
2932	GSK3B	glycogen synthase kinase 3 beta
841	CASP8	caspase 8, apoptosis-related cysteine peptidase
5925	RB1	retinoblastoma 1
5515	PPP2CA	protein phosphatase 2, catalytic subunit, alpha isozyme
3320	HSP90AA1	heat shock protein 90kDa alpha (cytosolic), class A member 1
2475	MTOR	mechanistic target of rapamycin (serine/threonine kinase)
1017	CDK2	cyclin-dependent kinase 2
3558	IL2	interleukin 2
4609	MYC	v-myc myelocytomatosis viral oncogene homolog (avian)
1499	CTNNB1	catenin (cadherin-associated protein), beta 1, 88kDa
6772	STAT1	signal transducer and activator of transcription 1, 91kDa
3717	JAK2	Janus kinase 2
598	BCL2L1	BCL2-like 1
355	FAS	Fas (TNF receptor superfamily, member 6)
472	ATM	ataxia telangiectasia mutated
3576	IL8	interleukin 8
3065	HDAC1	histone deacetylase 1
7422	VEGFA	vascular endothelial growth factor A
999	CDH1	cadherin 1, type 1, E-cadherin (epithelial)
3458	IFNG	interferon, gamma
3630	INS	insulin
2778	GNAS	GNAS complex locus
4851	NOTCH1	notch 1

5321	PLA2G4A	phospholipase A2, group IVA (cytosolic, calcium-dependent)
3569	IL6	interleukin 6 (interferon, beta 2)
2147	F2	coagulation factor II (thrombin)
4128	MAOA	monoamine oxidase A
993	CDC25A	cell division cycle 25 homolog A (S. pombe)
3553	IL1B	interleukin 1, beta
5580	PRKCD	protein kinase C, delta
2002	ELK1	ELK1, member of ETS oncogene family
1173	AP2M1	adaptor-related protein complex 2, mu 1 subunit
5608	MAP2K6	mitogen-activated protein kinase kinase 6
7465	WEE1	WEE1 homolog (S. pombe)
5347	PLK1	polo-like kinase 1
834	CASP1	caspase 1, apoptosis-related cysteine peptidase (interleukin 1, beta, convertase)
5045	FURIN	furin (paired basic amino acid cleaving enzyme)
1027	CDKN1B	cyclin-dependent kinase inhibitor 1B (p27, Kip1)
2805	GOT1	glutamic-oxaloacetic transaminase 1, soluble (aspartate aminotransferase 1)
1022	CDK7	cyclin-dependent kinase 7
3383	ICAM1	intercellular adhesion molecule 1
1576	CYP3A4	cytochrome P450, family 3, subfamily A, polypeptide 4
4089	SMAD4	SMAD family member 4
58	ACTA1	actin, alpha 1, skeletal muscle
1544	CYP1A2	cytochrome P450, family 1, subfamily A, polypeptide 2
3122	HLA-DRA	major histocompatibility complex, class II, DR alpha
2260	FGFR1	fibroblast growth factor receptor 1
3091	HIF1A	hypoxia inducible factor 1, alpha subunit (basic helix-loop-helix transcription factor)
5581	PRKCE	protein kinase C, epsilon
135	ADORA2A	adenosine A2a receptor
4193	MDM2	Mdm2 p53 binding protein homolog (mouse)
839	CASP6	caspase 6, apoptosis-related cysteine peptidase
2547	XRCC6	X-ray repair complementing defective repair in Chinese hamster cells 6
672	BRCA1	breast cancer 1, early onset
6722	SRF	serum response factor (c-fos serum response element-binding transcription factor)
351	APP	amyloid beta (A4) precursor protein
5155	PDGFB	platelet-derived growth factor beta polypeptide (simian sarcoma viral (v-sis) oncogene homolog)
3295	HSD17B4	hydroxysteroid (17-beta) dehydrogenase 4
3014	H2AFX	H2A histone family, member X
3654	IRAK1	interleukin-1 receptor-associated kinase 1
1020	CDK5	cyclin-dependent kinase 5

7852	CXCR4	chemokine (C-X-C motif) receptor 4
2247	FGF2	fibroblast growth factor 2 (basic)
1965	EIF2S1	eukaryotic translation initiation factor 2, subunit 1 alpha, 35kDa
7046	TGFBR1	transforming growth factor, beta receptor 1
5331	PLCB3	phospholipase C, beta 3 (phosphatidylinositol-specific)
5743	PTGS2	prostaglandin-endoperoxide synthase 2 (prostaglandin G/H synthase and cyclooxygenase)
3479	IGF1	insulin-like growth factor 1 (somatomedin C)
5328	PLAU	plasminogen activator, urokinase
5465	PPARA	peroxisome proliferator-activated receptor alpha
8648	NCOA1	nuclear receptor coactivator 1
4023	LPL	lipoprotein lipase
11168	PSIP1	PC4 and SFRS1 interacting protein 1
2064	ERBB2	v-erb-b2 erythroblastic leukemia viral oncogene homolog 2, neuro/glioblastoma derived oncogene homolog (avian)
3949	LDLR	low density lipoprotein receptor
6093	ROCK1	Rho-associated, coiled-coil containing protein kinase 1
5337	PLD1	phospholipase D1, phosphatidylcholine-specific
3661	IRF3	interferon regulatory factor 3
11343	MGLL	monoglyceride lipase
5243	ABCB1	ATP-binding cassette, sub-family B (MDR/TAP), member 1
1128	CHRM1	cholinergic receptor, muscarinic 1
5901	RAN	RAN, member RAS oncogene family
1571	CYP2E1	cytochrome P450, family 2, subfamily E, polypeptide 1
6531	SLC6A3	solute carrier family 6 (neurotransmitter transporter, dopamine), member 3
102	ADAM10	ADAM metallopeptidase domain 10
1109	AKR1C4	aldo-keto reductase family 1, member C4 (chlordecone reductase; 3-alpha hydroxysteroid dehydrogenase, type I; dihydrodiol dehydrogenase 4)
2626	GATA4	GATA binding protein 4
7341	SUMO1	SMT3 suppressor of mif two 3 homolog 1 (S. cerevisiae)
5329	PLAUR	plasminogen activator, urokinase receptor
408	ARRB1	arrestin, beta 1
25	ABL1	c-abl oncogene 1, non-receptor tyrosine kinase
2254	FGF9	fibroblast growth factor 9 (glia-activating factor)
3284	HSD3B2	hydroxy-delta-5-steroid dehydrogenase, 3 beta- and steroid delta-isomerase 2
2931	GSK3A	glycogen synthase kinase 3 alpha
2237	FEN1	flap structure-specific endonuclease 1
6915	TBXA2R	thromboxane A2 receptor
5562	PRKAA1	protein kinase, AMP-activated, alpha 1 catalytic subunit
60	ACTB	actin, beta
1719	DHFR	dihydrofolate reductase
7252	TSHB	thyroid stimulating hormone, beta

2908	NR3C1	nuclear receptor subfamily 3, group C, member 1 (glucocorticoid receptor)
2645	GCK	glucokinase (hexokinase 4)
1634	DCN	decorin
124	ADH1A	alcohol dehydrogenase 1A (class I), alpha polypeptide
7329	UBE2I	ubiquitin-conjugating enzyme E2I (UBC9 homolog, yeast)
150	ADRA2A	adrenergic, alpha-2A-, receptor
4190	MDH1	malate dehydrogenase 1, NAD (soluble)
8714	ABCC3	ATP-binding cassette, sub-family C (CFTR/MRP), member 3
27006	FGF22	fibroblast growth factor 22
6720	SREBF1	sterol regulatory element binding transcription factor 1
5160	PDHA1	pyruvate dehydrogenase (lipoamide) alpha 1
7498	XDH	xanthine dehydrogenase
2744	GLS	glutaminase
10714	POLD3	polymerase (DNA-directed), delta 3, accessory subunit
1548	CYP2A6	cytochrome P450, family 2, subfamily A, polypeptide 6
1573	CYP2J2	cytochrome P450, family 2, subfamily J, polypeptide 2
2678	GGT1	gamma-glutamyltransferase 1
1591	CYP24A1	cytochrome P450, family 24, subfamily A, polypeptide 1
3596	IL13	interleukin 13
43	ACHE	acetylcholinesterase
2194	FASN	fatty acid synthase
1136	CHRNA3	cholinergic receptor, nicotinic, alpha 3
1734	DIO2	deiodinase, iodothyronine, type II
1583	CYP11A1	cytochrome P450, family 11, subfamily A, polypeptide 1
3170	FOXA2	forkhead box A2
3098	HK1	hexokinase 1
2864	FFAR1	free fatty acid receptor 1
5584	PRKCI	protein kinase C, iota
9429	ABCG2	ATP-binding cassette, sub-family G (WHITE), member 2
6696	SPP1	secreted phosphoprotein 1
567	B2M	beta-2-microglobulin
2746	GLUD1	glutamate dehydrogenase 1
183	AGT	angiotensinogen (serpin peptidase inhibitor, clade A, member 8)
1675	CFD	complement factor D (adipsin)
3356	HTR2A	5-hydroxytryptamine (serotonin) receptor 2A
3158	HMGCS2	3-hydroxy-3-methylglutaryl-CoA synthase 2 (mitochondrial)
213	ALB	albumin
7222	TRPC3	transient receptor potential cation channel, subfamily C, member 3
1738	DLD	dihydrolipoamide dehydrogenase
10057	ABCC5	ATP-binding cassette, sub-family C (CFTR/MRP), member 5
6790	AURKA	aurora kinase A

229	ALDOB	aldolase B, fructose-bisphosphate
2902	GRIN1	glutamate receptor, ionotropic, N-methyl D-aspartate 1
2775	GNAO1	guanine nucleotide binding protein (G protein), alpha activating activity polypeptide O
2152	F3	coagulation factor III (thromboplastin, tissue factor)
7098	TLR3	toll-like receptor 3
2569	GABRR1	gamma-aminobutyric acid (GABA) receptor, rho 1
3758	KCNJ1	potassium inwardly-rectifying channel, subfamily J, member 1
5888	RAD51	RAD51 homolog (RecA homolog, E. coli) (S. cerevisiae)
10800	CYSLTR1	cysteinyl leukotriene receptor 1
1813	DRD2	dopamine receptor D2
2584	GALK1	galactokinase 1
3417	IDH1	isocitrate dehydrogenase 1 (NADP+), soluble
6609	SMPD1	sphingomyelin phosphodiesterase 1, acid lysosomal
2683	B4GALT1	UDP-Gal:betaGlcNAc beta 1,4- galactosyltransferase, polypeptide 1
1158	CKM	creatine kinase, muscle
3945	LDHB	lactate dehydrogenase B
3039	HBA1	hemoglobin, alpha 1
3309	HSPA5	heat shock 70kDa protein 5 (glucose-regulated protein, 78kDa)
1901	S1PR1	sphingosine-1-phosphate receptor 1
4351	MPI	mannose phosphate isomerase
3175	ONECUT1	one cut homeobox 1
644	BLVRA	biliverdin reductase A
3303	HSPA1A	heat shock 70kDa protein 1A
2554	GABRA1	gamma-aminobutyric acid (GABA) A receptor, alpha 1
5429	POLH	polymerase (DNA directed), eta
2492	FSHR	follicle stimulating hormone receptor
7018	TF	transferrin
22943	DKK1	dickkopf homolog 1 (Xenopus laevis)
3759	KCNJ2	potassium inwardly-rectifying channel, subfamily J, member 2
3312	HSPA8	heat shock 70kDa protein 8
11255	HRH3	histamine receptor H3
6528	SLC5A5	solute carrier family 5 (sodium iodide symporter), member 5
4904	YBX1	Y box binding protein 1
328	APEX1	APEX nuclease (multifunctional DNA repair enzyme) 1
2936	GSR	glutathione reductase
5034	P4HB	prolyl 4-hydroxylase, beta polypeptide
9734	HDAC9	histone deacetylase 9
10213	PSMD14	proteasome (prosome, macropain) 26S subunit, non-ATPase, 14
768	CA9	carbonic anhydrase IX
6822	SULT2A1	sulfotransferase family, cytosolic, 2A, dehydroepiandrosterone (DHEA)-preferring, member 1

554	AVPR2	arginine vasopressin receptor 2
1431	CS	citrate synthase
7846	TUBA1A	tubulin, alpha 1a
5593	PRKG2	protein kinase, cGMP-dependent, type II
7084	TK2	thymidine kinase 2, mitochondrial
5451	POU2F1	POU class 2 homeobox 1
10135	NAMPT	nicotinamide phosphoribosyltransferase
9446	GSTO1	glutathione S-transferase omega 1
5339	PLEC	plectin
1536	CYBB	cytochrome b-245, beta polypeptide
4967	OGDH	oxoglutarate (alpha-ketoglutarate) dehydrogenase (lipoamide)
383	ARG1	arginase, liver
6865	TACR2	tachykinin receptor 2
4684	NCAM1	neural cell adhesion molecule 1
8732	RNGTT	RNA guanylyltransferase and 5'-phosphatase
2030	SLC29A1	solute carrier family 29 (nucleoside transporters), member 1
130399	ACVR1C	activin A receptor, type IC
60482	SLC5A7	solute carrier family 5 (choline transporter), member 7
6389	SDHA	succinate dehydrogenase complex, subunit A, flavoprotein (Fp)
6950	TCP1	t-complex 1
6558	SLC12A2	solute carrier family 12 (sodium/potassium/chloride transporters), member 2
26986	PABPC1	poly(A) binding protein, cytoplasmic 1
7097	TLR2	toll-like receptor 2
2911	GRM1	glutamate receptor, metabotropic 1
5587	PRKD1	protein kinase D1
2717	GLA	galactosidase, alpha
782	CACNB1	calcium channel, voltage-dependent, beta 1 subunit
522	ATP5J	ATP synthase, H+ transporting, mitochondrial Fo complex, subunit F6
410	ARSA	arylsulfatase A
796	CALCA	calcitonin-related polypeptide alpha
59	ACTA2	actin, alpha 2, smooth muscle, aorta
4784	NFIX	nuclear factor I/X (CCAAT-binding transcription factor)
162466	PHOSPHO1	phosphatase, orphan 1
2023	ENO1	enolase 1, (alpha)
59350	RXFP1	relaxin/insulin-like family peptide receptor 1
392	ARHGAP1	Rho GTPase activating protein 1
3777	KCNK3	potassium channel, subfamily K, member 3
3061	HCRTR1	hypocretin (orexin) receptor 1
4669	NAGLU	N-acetylglucosaminidase, alpha
55825	PECR	peroxisomal trans-2-enoyl-CoA reductase
2098	ESD	esterase D

178	AGL	amylo-alpha-1, 6-glucosidase, 4-alpha-glucanotransferase
3783	KCNN4	potassium intermediate/small conductance calcium-activated channel, subfamily N, member 4
2923	PDIA3	protein disulfide isomerase family A, member 3
221391	OPN5	opsin 5
2629	GBA	glucosidase, beta, acid
4153	MBL2	mannose-binding lectin (protein C) 2, soluble
92935	MARS2	methionyl-tRNA synthetase 2, mitochondrial
4582	MUC1	mucin 1, cell surface associated
716	C1S	complement component 1, s subcomponent
9132	KCNQ4	potassium voltage-gated channel, KQT-like subfamily, member 4
52	ACP1	acid phosphatase 1, soluble
6606	SMN1	survival of motor neuron 1, telomeric
801	CALM1	calmodulin 1 (phosphorylase kinase, delta)
920	CD4	CD4 molecule
107	ADCY1	adenylate cyclase 1 (brain)
2782	GNB1	guanine nucleotide binding protein (G protein), beta polypeptide 1
6117	RPA1	replication protein A1, 70kDa
3030	HADHA	hydroxyacyl-CoA dehydrogenase/3-ketoacyl-CoA thiolase/enoyl-CoA hydratase (trifunctional protein), alpha subunit
4846	NOS3	nitric oxide synthase 3 (endothelial cell)
919	CD247	CD247 molecule
5829	PXN	paxillin
38	ACAT1	acetyl-CoA acetyltransferase 1
7504	XK	X-linked Kx blood group (McLeod syndrome)
1892	ECHS1	enoyl CoA hydratase, short chain, 1, mitochondrial
2697	GJA1	gap junction protein, alpha 1, 43kDa
718	C3	complement component 3
1977	EIF4E	eukaryotic translation initiation factor 4E
293	SLC25A6	solute carrier family 25 (mitochondrial carrier; adenine nucleotide translocator), member 6
6470	SHMT1	serine hydroxymethyltransferase 1 (soluble)
6898	TAT	tyrosine aminotransferase
5563	PRKAA2	protein kinase, AMP-activated, alpha 2 catalytic subunit
6582	SLC22A2	solute carrier family 22 (organic cation transporter), member 2
5610	EIF2AK2	eukaryotic translation initiation factor 2-alpha kinase 2
6844	VAMP2	vesicle-associated membrane protein 2 (synaptobrevin 2)
7450	VWF	von Willebrand factor
501	ALDH7A1	aldehyde dehydrogenase 7 family, member A1
2966	GTF2H2	general transcription factor IIH, polypeptide 2, 44kDa
8106	PABPN1	poly(A) binding protein, nuclear 1
2328	FMO3	flavin containing monooxygenase 3

9965	FGF19	fibroblast growth factor 19
1644	DDC	dopa decarboxylase (aromatic L-amino acid decarboxylase)
1374	CPT1A	carnitine palmitoyltransferase 1A (liver)
54658	UGT1A1	UDP glucuronosyltransferase 1 family, polypeptide A1
8818	DPM2	dolichyl-phosphate mannosyltransferase polypeptide 2, regulatory subunit
55902	ACSS2	acyl-CoA synthetase short-chain family member 2
2891	GRIA2	glutamate receptor, ionotropic, AMPA 2
727	C5	complement component 5
10905	MAN1A2	mannosidase, alpha, class 1A, member 2
4998	ORC1	origin recognition complex, subunit 1
2784	GNB3	guanine nucleotide binding protein (G protein), beta polypeptide 3
7360	UGP2	UDP-glucose pyrophosphorylase 2
5422	POLA1	polymerase (DNA directed), alpha 1, catalytic subunit
6916	TBXAS1	thromboxane A synthase 1 (platelet)
6513	SLC2A1	solute carrier family 2 (facilitated glucose transporter), member 1
7321	UBE2D1	ubiquitin-conjugating enzyme E2D 1 (UBC4/5 homolog, yeast)
5440	POLR2K	polymerase (RNA) II (DNA directed) polypeptide K, 7.0kDa
375	ARF1	ADP-ribosylation factor 1
3073	HEXA	hexosaminidase A (alpha polypeptide)
1119	CHKA	choline kinase alpha
6091	ROBO1	roundabout, axon guidance receptor, homolog 1 (Drosophila)
11001	SLC27A2	solute carrier family 27 (fatty acid transporter), member 2
3655	ITGA6	integrin, alpha 6
9150	CTDP1	CTD (carboxy-terminal domain, RNA polymerase II, polypeptide A) phosphatase, subunit 1
10249	GLYAT	glycine-N-acyltransferase
1742	DLG4	discs, large homolog 4 (Drosophila)
1610	DAO	D-amino-acid oxidase
29968	PSAT1	phosphoserine aminotransferase 1
2817	GPC1	glypican 1
51166	AADAT	aminoadipate aminotransferase
1468	SLC25A10	solute carrier family 25 (mitochondrial carrier; dicarboxylate transporter), member 10
5167	ENPP1	ectonucleotide pyrophosphatase/phosphodiesterase 1
103	ADAR	adenosine deaminase, RNA-specific
22916	NCBP2	nuclear cap binding protein subunit 2, 20kDa
55300	PI4K2B	phosphatidylinositol 4-kinase type 2 beta
23169	SLC35D1	solute carrier family 35 (UDP-glucuronic acid/UDP-N-acetylgalactosamine dual transporter), member D1
142680	SLC34A3	solute carrier family 34 (sodium phosphate), member 3
4057	LTF	lactotransferrin
6507	SLC1A3	solute carrier family 1 (glial high affinity glutamate transporter), member 3

7015	TERT	telomerase reverse transcriptase
64240	ABCG5	ATP-binding cassette, sub-family G (WHITE), member 5
4968	OGG1	8-oxoguanine DNA glycosylase
286	ANK1	ankyrin 1, erythrocytic
6817	SULT1A1	sulfotransferase family, cytosolic, 1A, phenol-preferring, member 1
6005	RHAG	Rh-associated glycoprotein
6539	SLC6A12	solute carrier family 6 (neurotransmitter transporter, betaine/GABA), member 12
4122	MAN2A2	mannosidase, alpha, class 2A, member 2
8884	SLC5A6	solute carrier family 5 (sodium-dependent vitamin transporter), member 6
5406	PNLIP	pancreatic lipase
6482	ST3GAL1	ST3 beta-galactoside alpha-2,3-sialyltransferase 1
23539	SLC16A8	solute carrier family 16, member 8 (monocarboxylic acid transporter 3)
3776	KCNK2	potassium channel, subfamily K, member 2
84515	MCM8	minichromosome maintenance complex component 8
50674	NEUROG3	neurogenin 3
488	ATP2A2	ATPase, Ca++ transporting, cardiac muscle, slow twitch 2
8443	GNPAT	glyceronephosphate O-acyltransferase
8898	MTMR2	myotubularin related protein 2
2673	GFPT1	glutamine--fructose-6-phosphate transaminase 1
7351	UCP2	uncoupling protein 2 (mitochondrial, proton carrier)
6548	SLC9A1	solute carrier family 9 (sodium/hydrogen exchanger), member 1
6996	TDG	thymine-DNA glycosylase
376497	SLC27A1	solute carrier family 27 (fatty acid transporter), member 1
1915	EEF1A1	eukaryotic translation elongation factor 1 alpha 1
27010	TPK1	thiamin pyrophosphokinase 1
161823	ADAL	adenosine deaminase-like
6518	SLC2A5	solute carrier family 2 (facilitated glucose/fructose transporter), member 5
27161	EIF2C2	eukaryotic translation initiation factor 2C, 2
6523	SLC5A1	solute carrier family 5 (sodium/glucose cotransporter), member 1
4594	MUT	methylmalonyl CoA mutase
64902	AGXT2	alanine--glyoxylate aminotransferase 2
60490	PPCDC	phosphopantothenoylcysteine decarboxylase
6833	ABCC8	ATP-binding cassette, sub-family C (CFTR/MRP), member 8
760	CA2	carbonic anhydrase II
7298	TYMS	thymidylate synthetase
9695	EDEM1	ER degradation enhancer, mannosidase alpha-like 1
114134	SLC2A13	solute carrier family 2 (facilitated glucose transporter), member 13
211	ALAS1	aminolevulinate, delta-, synthase 1
6546	SLC8A1	solute carrier family 8 (sodium/calcium exchanger), member 1
7019	TFAM	transcription factor A, mitochondrial
4255	MGMT	O-6-methylguanine-DNA methyltransferase

51128	SAR1B	SAR1 homolog B (S. cerevisiae)
64816	CYP3A43	cytochrome P450, family 3, subfamily A, polypeptide 43
11019	LIAS	lipoic acid synthetase
10585	POMT1	protein-O-mannosyltransferase 1
3067	HDC	histidine decarboxylase
57369	GJD2	gap junction protein, delta 2, 36kDa
6628	SNRPB	small nuclear ribonucleoprotein polypeptides B and B1
9054	NFS1	NFS1 nitrogen fixation 1 homolog (S. cerevisiae)
5393	EXOSC9	exosome component 9
64849	SLC13A3	solute carrier family 13 (sodium-dependent dicarboxylate transporter), member 3
5819	PVRL2	poliovirus receptor-related 2 (herpesvirus entry mediator B)
64924	SLC30A5	solute carrier family 30 (zinc transporter), member 5
6521	SLC4A1	solute carrier family 4, anion exchanger, member 1 (erythrocyte membrane protein band 3, Diego blood group)
6565	SLC15A2	solute carrier family 15 (H+/peptide transporter), member 2
167227	DCP2	DCP2 decapping enzyme homolog (S. cerevisiae)
358	AQP1	aquaporin 1 (Colton blood group)
610	HCN2	hyperpolarization activated cyclic nucleotide-gated potassium channel 2
10058	ABCB6	ATP-binding cassette, sub-family B (MDR/TAP), member 6
50515	CHST11	carbohydrate (chondroitin 4) sulfotransferase 11
27173	SLC39A1	solute carrier family 39 (zinc transporter), member 1
29127	RACGAP1	Rac GTPase activating protein 1
7965	AIMP2	aminoacyl tRNA synthetase complex-interacting multifunctional protein 2
8972	MGAM	maltase-glucoamylase (alpha-glucosidase)
5471	PPAT	phosphoribosyl pyrophosphate amidotransferase
11285	B4GALT7	xylosylprotein beta 1,4-galactosyltransferase, polypeptide 7 (galactosyltransferase I)
115111	SLC26A7	solute carrier family 26, member 7

Table S2. Pathway assays that screened the Tox21 pilot phase collection of 2,870 compounds

Assay Target Pathway	Assay Readout	Target Gene
AhR	Luciferase	AHR
ARE	BLA	NFE2L2
Cell signaling CREB	BLA	CREB1
ESRE	BLA	ERN1, HERPUD1
Heat shock response	BLA	HSPB1
Heat shock response	Luciferase	HSPB1
HRE	BLA	HIF1A
IL-8	BLA	IL8
NFkB	BLA	NFKB1
AR	BLA	AR
Erα	BLA	ESR1
FXR	BLA	NR1H4
GR	BLA	NR3C1
LXR	BLA	NR1H2
PPARα	BLA	PPARA
PPARδ	BLA	PPARD
PPARγ	BLA	PPARG
PPARγ	Luciferase	PPARG
RXR	BLA	RXRA
TRβ	BLA	THRB
VDR	BLA	VDR
P53	BLA	TP53
PXR	Luciferase	NR1I2
PXR-P450	Luciferase	NR1I2, CYP3A4
TNFα	HTRF	TNF

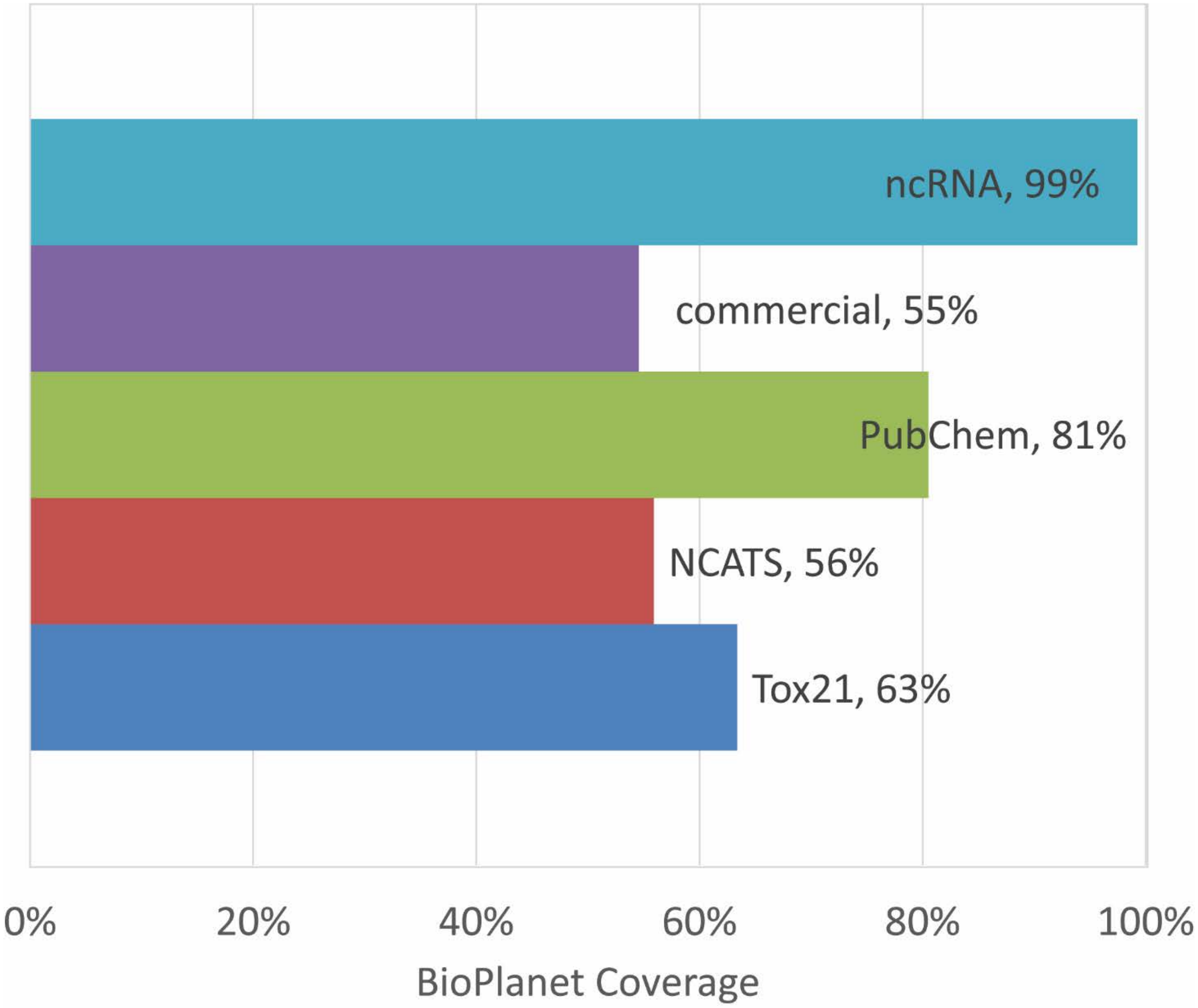

Figure S1. Coverage of pathways by different assay sources and ncRNA.

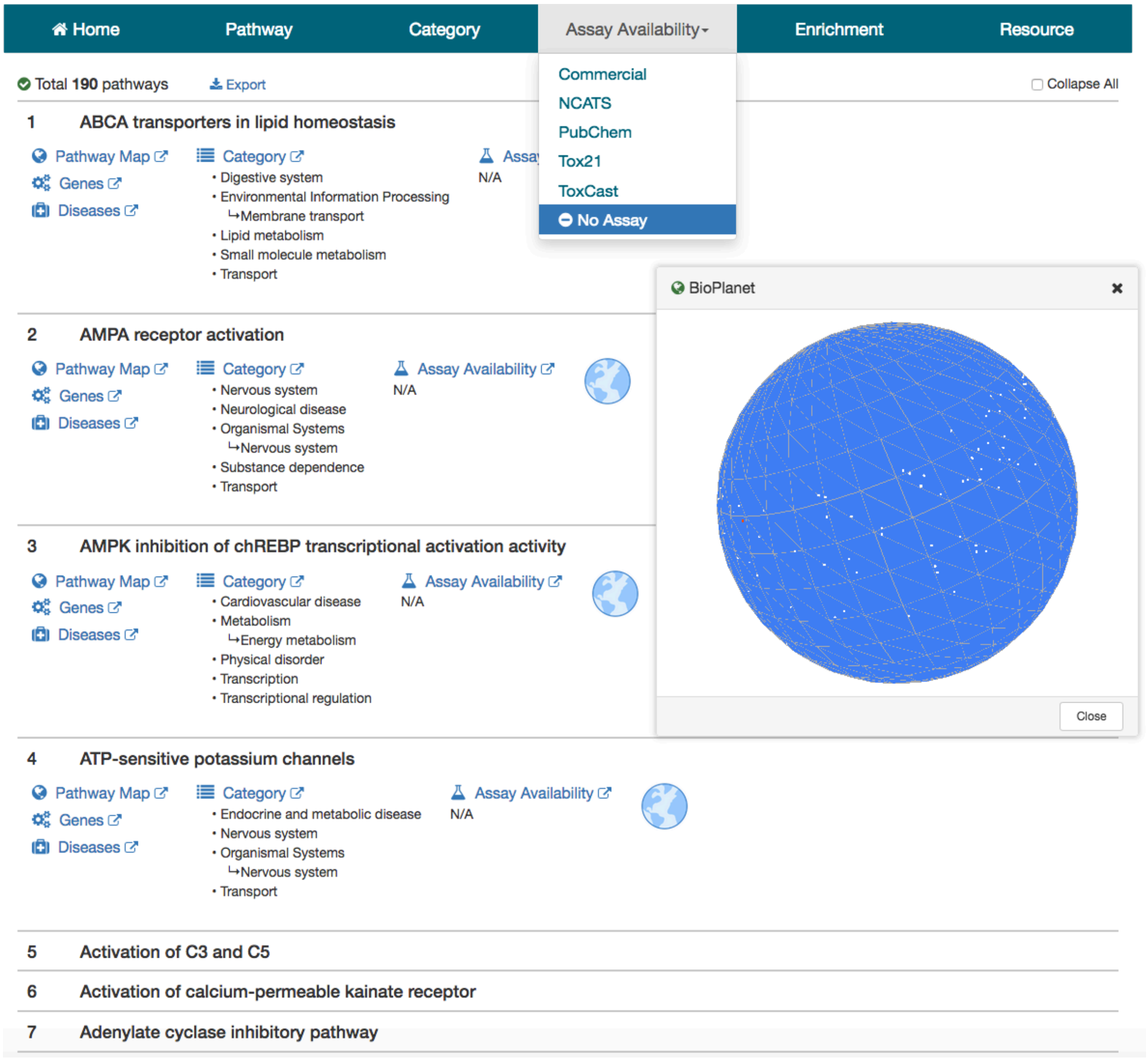

Figure S2. BioPlanet pathways that do not have a probing bioassay available. This list of pathways can be viewed and downloaded from the NCATS BioPlanet web browser.
